# *Enteromorpha prolifera* soluble dietary fiber alleviates ulcerative colitis through restoration of mucosal barrier and gut microbiota homeostasis

**DOI:** 10.3389/fnut.2025.1579889

**Published:** 2025-04-24

**Authors:** Yuan-Yuan Ding, Xin-Yi Tang, Yu Tian, Feng-Long Zhang, Xiang Ding, Meng-Chun Qi, Wei Dong, Chen-Guang Liu

**Affiliations:** ^1^College of Marine Life Sciences, Ocean University of China, Qingdao, China; ^2^College of Stomatology, North China University of Science and Technology, Tangshan, China

**Keywords:** *Enteromorpha prolifera*, dietary supplement, ulcerative colitis, gut microbiota, gut barrier

## Abstract

**Background:**

Ulcerative colitis (UC), a recurrent chronic colon inflammation, presents substantial therapeutic challenges due to the frequent adverse effects associated with conventional pharmacological treatments. These limitations underscore the critical need for developing alternative dietary interventions with improved safety profiles. The present study investigated the therapeutic potential of *Enteromorpha prolifera* soluble dietary fiber microparticles (EDFM) in UC management, focusing on restoring mucosal barrier integrity and modulating gut microbiota homeostasis.

**Methods:**

EDFM was fabricated through aqueous extraction of *E. prolifera* soluble dietary fiber via boiling followed by spray-drying. A mouse UC model was induced by dextran sulfate sodium (DSS). The severity of UC was evaluated through daily disease activity index (DAI) scoring; quantification of pro-inflammatory cytokines (TNF-*α*, IL-1β) via ELISA; histopathological analysis of colon sections with H&E staining; immunofluorescence detection of tight junction proteins (ZO-1, occludin); and 16S rRNA sequencing for gut microbiota.

**Results:**

EDFM treatment significantly reduced the expression of pro-inflammatory cytokines (TNF-*α* and IL-1β), enhanced the expression of tight junction proteins (ZO-1 and occludin), and stimulated mucin (MUC2) production. Additionally, EDFM promoted the proliferation of beneficial probiotics (*Alloprevotella, Lachnospiraceae_NK4A136_group,* and *Ruminococcaceae_UCG-014*), while inhibiting pathogenic bacteria (*Escherichia-Shigella, Parabacteroides*, *Rikenellaceae_RC9_gut_group, Odoribacter*, and *[Ruminococcus]_torques_group*).

**Conclusion:**

EDFM supplementation significantly ameliorates UC through dual modulation of gut microbiota and intestinal barrier integrity, indicating its potential as a functional food ingredient for UC prevention and treatment.

## 1 Introduction

Ulcerative colitis (UC), an inflammatory bowel disease, is a recurrent chronic inflammation of the colon (1). In recent decades, the prevalence and incidence of UC have notably increased, likely due to changes in lifestyle and environmental factors (2). During the 20th century, over 1.5 million North American and 2 million European are suffering from the disease (3). UC is mainly characterized by diarrhea, hematochezia, of intestinal barrier function impairment, and gut microbiota dysbiosis (4). Significantly, UC may increase the risk of colorectal cancer (5). Patients who experience a disease trajectory lasting longer than 20 years have a 10–15 times higher risk of colon cancer than ordinary people (5). Although the etiology of UC remains uncertain, various factors have been identified as potential causes of its pathogenesis and development, including host genetic susceptibility, environmental factors, the host immune system, gut microbiota and the integrity of the intestinal barrier (6).

The intestinal barrier plays a crucial role in the health of humans and animals. The intestinal barrier comprises physical, chemical, immune and biological barrier (7). As the intestinal physical barrier, intestinal epithelial cells depend on the precise organization of intercellular and intracellular tight junction proteins, including ZO-1 and occludin, to establish the structural integrity of the intestinal barrier (1). The intestinal physical barrier can impede the passage of toxins, germs, viruses, and other deleterious substances, while simultaneously facilitating the transit of advantageous substances (8). Intestinal mucus serves as the intestinal chemical barrier, maintaining intestinal homeostasis, protecting intestinal epithelial cells from physical, chemical, and biological damage, and creating a favorable habitat and nourishment for symbiotic bacteria residing in the gut (9). Gut microbiota, served as intestinal biological barrier, can modulate the host’s immune response, suppress the proliferation of intestinal pathogens, modulate intestinal homeostasis, and maintain intestinal barrier integrity (1, 10). For UC patients, their intestinal barriers are impaired, manifested as damaged epithelial tight junctions (11), decreased intestinal mucus (12), and dysregulated gut microbiota (13). In contrast, the disrupt of intestinal barrier exacerbates inflammation and even directly induces UC (14). Therefore, the restoration of the integrity of the intestinal barrier is beneficial for the anesis of UC.

Currently, there is no curative treatment for UC. The primary objective of existing treatments is to manage and alleviate the patient’s symptoms rather than cure the disease. Treatment for UC patients often spans a lifetime, with pharmaceutical interventions being the primary method. However, prolonged pharmaceutical interventions may result in serious side effects and drug resistance, such as vomiting, fatigue, diarrhea, and abdominal pain (15). Researchers have made significant efforts to explore novel treatment strategies for UC, including oral probiotics, fecal microbiota transplantation (FMT), and dietary fiber supplements. However, the effectiveness of oral probiotics is hindered by their weak resistance to gastric acid and poor intestinal colonization ability. FMT is constrained by the availability of medical equipment and skilled personnel, limiting its use to only a small number of UC patients. Dietary fiber is an edible plant component resistant to digestion and absorption in the small intestinal area, partially or completely fermented in the colon by gut microbiota (16). Studies have demonstrated the beneficial effects of dietary fiber on the immune system, intestinal mucosal repair, gut microbiota regulation, and the occurrence of bacterial translocation (17). Notably, the absence of dietary fiber can result in elevated intestinal permeability, a reduction in mucus thickness and dysregulated gut microbiota, which is prone to inducing UC (18, 19). This demonstrates that the supplement of dietary fiber in the diet may potentially restore the integrity of the intestinal barrier, thus preventing and treating UC.

Dietary fiber is commonly categorized as soluble dietary fiber and insoluble dietary fiber based on its solubility in water (20). And it is now widely recognized that the health-promoting effects of the dietary fiber depend on its source, structure and composition. Dietary fiber can be naturally obtained from both terrestrial plants and marine seaweeds. Seaweeds, in particular, are rich in dietary fiber and offer a higher nutritional value when compared to terrestrial plants (17). *Enteromorpha prolifera* (*E. prolifera*) is an edible green seaweed used worldwide in the food and medical industry, rich in soluble dietary fiber (21). Wherein polysaccharides are the primary constituent of utmost significance. The polysaccharides from *E. prolifera* (EP) are considered as a safe compound with various physiological functions, such as antioxidant (22), anti-inflammatory (23), immunomodulatory (24), tissue restoration (25), and gut microbiota modulatory (26) properties. These effects are attributed to its unique structure of sulfated rhamnose. According to previous researches, it has been proved that EP exhibits preventative and mitigating properties concerning several diseases, including pancreatic damage (27), lipid metabolism disorders (28), excessive obesity (29), and intestinal inflammation (30). The soluble dietary fiber extracted from *E. prolifera* not only contains a high content of EP but also includes water-soluble proteins and minerals, all of which exert beneficial effects on human health (31, 32). In addition, uniform micro/nanoparticles have high contact area with intestinal epithelial cells, thereby enhancing bioavailability. Simultaneously, they are conducive to the adhesion and fermentation by gut microbiota, thus amplifying their prebiotic effects (1). Therefore, *E. prolifera* dietary fiber micro/nanoparticle (EDFM) prepared using spray-drying not only maintain the stability of active components but also optimize their functional performance, representing a promising functional food formulation with significant application potential. Although the activity of EP in alleviating inflammation and regulating gut microbiota has been fully demonstrated, it is still unclear whether EDFM can be used as a dietary supplement to promote the repair of intestinal barrier function, thereby alleviating UC.

Herein, EDFM might be used for UC treatment as a dietary supplement. The unique sulfated polysaccharide in EDFM exhibit anti-inflammatory, immunomodulatory, and microbiota-regulatory properties, suggesting its potential to restore intestinal barrier integrity in UC. Moreover, the micro/nanoparticle formulation of EDFM could enhance its interaction with epithelial cells and gut microbiota, potentially amplifying its therapeutic effects. This study will present an experimental foundation supporting the utilization of EDFM as a functional component in a specialized medicinal nutrition product designed to prevent and treat UC.

## 2 Materials and methods

### 2.1 Materials and reagents

Fresh *E. prolifra* was collected from the coast of Qingdao (36°10′N; 120°47′E), China. Fresh *E. prolifra* was washed with tap water, followed by removing excess water. Then the above clean *E. prolifra* was dried by freeze dryer (SCIENTZ-10 N, Ningbo Xinzhi Biotechnology Co., Ltd., China). Mannose (Man), Ribose (Rib), Rhamnose (Rha), Glucuronic acid (GluA), Glucose (Glc), Xylose (Xyl), Arabinose (Ara), and DSS (35–50 kDa) were purchased from Sigma Chemical Co. (St. Louis, MO, USA). Antibodies against zonula occludens-1 (ZO-1) and occludin were purchased from Abcam (Cambridge, UK). Mesalazine enteric-coated tablets were purchased from Losan Pharma GmbH (Neuenburg, Germany). Absolute ethanol, Sulphuric acid, BCA protein quantitative kit, Anthrone reagent, Acetone, and Methanol were obtained from Sinopharm Chemical Reagent Co., Ltd. (Shanghai, China). Mouse Myeloperoxidase (MPO) activity assay kit, TNF-*α* and IL-1β enzyme-linked immunosorbent assay (ELISA) kits were obtained from R&D Systems China (Shanghai, China). Hematoxylin and eosin staining kit, fluorescent secondary antibodies and 4′, 6-diamidino-2-phe-nylindole (DAPI) were purchased from Beijing Solarbio Technology Co., Ltd. (Beijing, China).

### 2.2 Nutritional composition analysis of *E. prolifra*

Moisture content, ash content, water-soluble polysaccharides content and crude fiber content of *E. prolifra* were determined according to the methods previously reported research (33). Protein and fat contents were determined by Kjeldahl method (34) and soxhlet extractor method according to previously reported with some modifications (35), respectively.

### 2.3 Preparation of *E. prolifera* soluble dietary fiber microparticles

**Figure 1:**
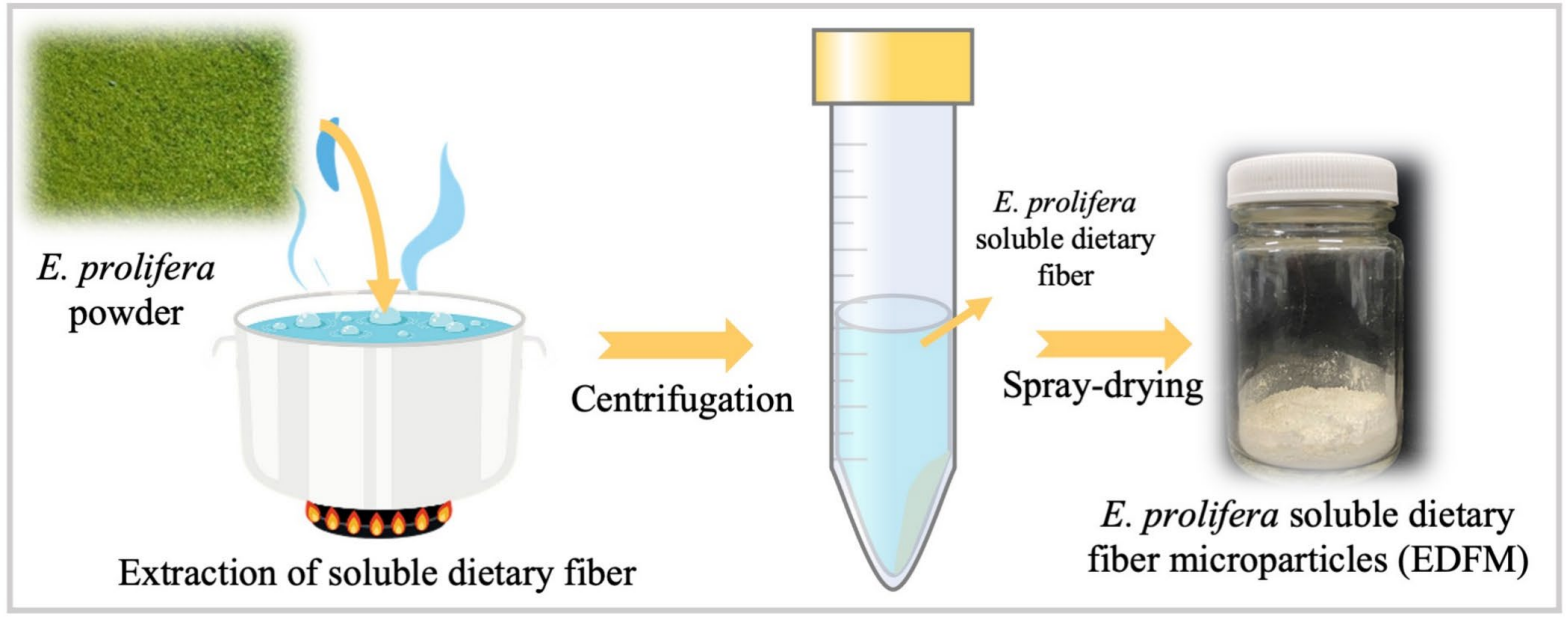
Flowchart of EDFM preparation.

The flowchart of *E. prolifera* soluble dietary fiber microparticle (EDFM) preparation was showed in Figure 1. Specifically, 12 g *E. prolifra* and 960 mL distilled water were placed in a vacuum wall breaker, then breaking at vacuum condition for 5 min. Further, the above mixture was boiled for 3 h, followed by centrifugation at 8000 *g* for 10 min. Then, the supernatant and precipitation were collected, respectively. The boiling process was performed twice. Finally, the supernatant was concentrated to 400 mL (36, 37).

EDFM was manufactured by a spray dryer (B-290, Buchi, Switzerland), according to the reported study (38). The preparation process of EDFM was optimized by single factor analysis using its yield and particle size as a criterion. Firstly, the air inlet volume, air pressure and injection speed were set to 100%, 40 mm and 45%, respectively. The inlet temperature was optimized by adjusting it to 120°C, 140°C, 160°C, 180°C and 200°C. Secondly, the air inlet volume, air pressure and inlet temperature were set to 100%, 40 mm and 180°C, respectively. The injection speed was optimized by adjusting it to 25, 35, 45, 55, and 60%.

### 2.4 Characterization of EDFM

The particle size and particle size distribution were characterized by a micro nano laser particle sizer (Winner 2,000, Jinan, China). The morphology of EDFM was observed by optical microscope (DSX1000, Olympus, Japan), and its particle size was counted with Nanomeasure 1.2 software. Moisture, ash, protein, fat, and polysaccharide contents of EDFM were determined according to previously reported methods (33). The polysaccharides were separated from EDFM. Briefly, EDFM was dissolved into distilled water (15 mg/mL), and then the EDFM solution was mixed with alcohol at a volume ratio 1:3, staying overnight. Next, the precipitation was collected and dried at 50°C for 5 h to get the crude polysaccharide. Subsequently, the crude polysaccharide was purified by chromatography of Q Sepharose FF column (39). The polysaccharide solution (5 mg/mL, 4 mL) was applied to a column (3 cm × 20 cm). Then, the column was eluted by stepwise elution of NaCl solution at a 2 mL/min flow rate. The eluates were collected and then analysed using the phenol-sulfuric acid method. The eluates from the same peak were collected and dialyszed to remove NaCl, then concentrated and lyophilized to obtain the purified polysaccharide. Furthermore, the molecular weight was analyzed by Gel Permeation Chromatography (GPC). The sulfate content was determined using the barium chloride-gelatin method, as described in previous studies (40). Finally, the monosaccharide composition was analyzed by 1-phenyl-3-methyl-5-pyrazolone (PMP) precolumn derivation high performance liquid chromatography (HPLC) according to previously reported study (40).

### 2.5 Induction and EDFM treatment of DSS-induced UC

The induction and EDFM dietary treatment regimen of DSS-induced UC was shown in Supplementary Figure S1. Briefly, sixty C57BL/6 mice (Male, 20 ± 2 g, 6 weeks old) were purchased from Vital River Laboratories (Beijing, China). All mice were housed in standard polycarbonate cages with sterile bedding and maintained in a controlled environment (25°C, 55% relative humidity) with adequate ventilation. They were allowed to acclimate for 7 days prior to the experiment. During the adaptive feeding period, the mice were fed with AIN-9G. Then the mice were randomly divided into 6 groups [Healthy group (Healthy); UC group (UC), Mesalazine-treated group (Mesalazine); low-dosed EDFM group (LEDFM); Medium-dosed EDFM group (MEDFM); High-dosed EDFM group (HEDFM)] according to their body weight (*n* = 10 per group). Before inducing UC, the mice in the Healthy, UC and Mesalazine groups were fed with AIN-93G for 15 days. The mice in the LEDFM, MEDFM and HEDFM groups were fed with AIN-9G supplemented with 19 mg EDFM /100 g AIN-93G, 38 mg EDFM /100 g AIN-93G and 96 mg EDFM/100 g AIN-93G for 15 days, respectively. Next, the mice of all experimental groups except for the Healthy group were intragastrically administered with DSS of 4 g/kg**·**d for 5 days to induce UC. After inducing UC, the mice in the Mesalazine group were intragastrically administered with Mesalazine enteric-coated tablets (614 mg/kg), while others were intragastrically administered physiological saline. The kind of diet for each group remained unchanged throughout the entire experimental process. Each mouse was fed with 20 g of experimental diet every day, and the daily food intake of mice was monitored. All animal experiments were performed with the approval of the Experimental Animal Ethics Committee of Ocean University of China (accreditation number: OUC-AE-2023-154).

### 2.6 Measurements of disease activity index (DAI)

The body weight, stool characteristics and fecal occult blood of mice were recorded daily. According to Supplementary Table S1, the scores of the body weight, stool characteristics and fecal occult blood of each mouse were scored. Further, based on the standards reported by Jin et al. (41), the DAI values were calculated using the following equation.


$$\begin{array}{l} DAI=(\mathrm{body}\;\mathrm{weight}\;\mathrm{loss}\;\mathrm{score}+\mathrm{stool}\;\mathrm{characters}\;\mathrm{score}\\ +\mathrm{fecal}\;\mathrm{occult}\;\mathrm{blood})/3 \end{array}$$

### 2.7 Collection and processing of blood samples, spleen and colon tissue of mice

The day before the end of the experiment, all mice were fasted for one night with only access to water. The blood samples of mice were obtained by removing eyes and centrifuged at 4°C, 3000 *g* for 10 min, and then the upper serum was taken and stored at −80°C for cytokine measurements. Further, the mice were sacrificed by cervical dislocation, and the colon and spleen tissues were harvested and weighed. The length of the colon was also measured. In addition, the colon tissue was divided into two parts: one part was fixed in 4% paraformaldehyde, and the other one was homogenized by tissue homogenizer (F6/10, Shanghai Jingxin Industrial Development Co., Ltd., China). Then, the colon homogenate was centrifugated and the supernatant was obtained to store at −80°C for cytokine measurements.

### 2.8 Cytokine assay

The levels of cytokines of TNF-*α* and IL-1β in serum and colon tissues were measured by ELISA kits according to the manufacturer’s protocols.

### 2.9 Histological and immunofluorescence analyses

The colon tissues fixed in 4% paraformaldehyde were embedded in paraffin, and sliced into 4 μm sections. These slices of the colon were stained with hematoxylin and eosin (H&E) and alcian blue and periodic acid-Schiff (AB-PAS) (42) and subsequently observed with a light microscope (DSX1,000, Olympus, Japan). The histological scores of H&E-stained sections were performed using the evaluation criteria showed in Supplementary Table S2 reported by Xu et al. (42).

For immunofluorescence analysis, the slices of the colon were dewaxed, rehabilitated and incubated antibodies (ZO-1 and occludin, 1:500) at 4°C overnight. The slices were then incubated with CY3 and FITC-labeled secondary antibodies and treated with DAPI for nuclear counterstaining. The slices were scanned with a 3DHISTECH Pannoramic MIDI digital slide scanner and analyzed using the CaseViewer software. The mean densities of ZO-1 and occludin were analyzed using the ImageJ software (43).

### 2.10 Gut microbiota 16S sequencing assay

On the final day of the experiment, fecal samples aseptically collected from 6 randomly selected mice per group (healthy, UC, and HEDFM groups) during the diurnal inactive phase (09:00–11:00) to standardize circadian rhythm effects. Immediately upon collection, samples were frozen in liquid nitrogen, and sent to Biomarker Technologies, Inc. (Beijing, China) for gut microbiota analysis by 16S sequencing assay. Briefly, microbiome DNA was extracted from the feces of the mouse by a QIAamp DNA Stool Mini Kit (Germantown, MD, United States) and the 16S rRNA libraries were constructed using the VAHTS Universal DNA Library Prep Kit for Illumina on an Illumina MiSeq (Illumina, Novaseq 6,000). A panel of GENEWIZ’s proprietary primers containing the sequences “CCTACGGRRBGCASCAGKVRVGAAT” (forward primers) and “GGACTACNVGGGTWTCTAATCC” (reverse primers) were adopted for the construction, which was specific to the V3-V4 regions of the microbiota 16S rRNA.

16S rRNA gene sequencing analysis was performed using the QIIME 2 data analysis package. Specifically, the forward and reverse reads were linked and allotted to samples according to the barcode, and then the barcode and primer sequence were further removed. The obtained product was filtered to delete the sequences that contained ambiguous bases, whose length was over 200 bp, or whose mean quality score was less than 20. The chimeric sequences were determined by a reference database (RDP Gold database) with the UCHIME algorithm and discarded to obtain the effective sequences for ultimate analysis. The clustering program VSEARCH (1.9.6) was utilized to cluster the sequences into operational taxonomic units (OTUs) with 97% sequence identity. The 16S rRNA reference database was Silva 132, and taxonomic category analysis was performed on all OTUs with an 80% confidence threshold using the Ribosomal Database Program (RDP) classifier. Finally, the Chao1, Shannon, Simpson, ACE, and beta diversity index was calculated in the QIIME 2 data analysis package according to the OTU analysis results. For microbial correlation analysis, Spearman’s rank correlation was performed to assess relationships between gut microbiota composition and UC-related parameters. Heatmaps were generated to visualize the relative abundance of microbial taxa and their correlations with clinical indicators. The linear discriminant analysis (LDA) and LDA effect size (LEfSe) methods (the threshold of a logarithmic score of LDA analysis was set to 4.0) were applied to analyze the metagenomic biomarker among groups using the Galaxy Online Analysis Platform.

### 2.11 Statistical analysis

The experiments *in vitro* were conducted in triplicate (*n* = 3) and *in vivo* were conducted in sextuplicate (*n* = 6). The results are presented as the mean ± standard deviation. Comparative analysis of means was conducted using one-way ANOVA, with statistical significance set at *p* < 0.05.

## 3 Results

### 3.1 EDFM prepared by spray drying

**Figure 2:**
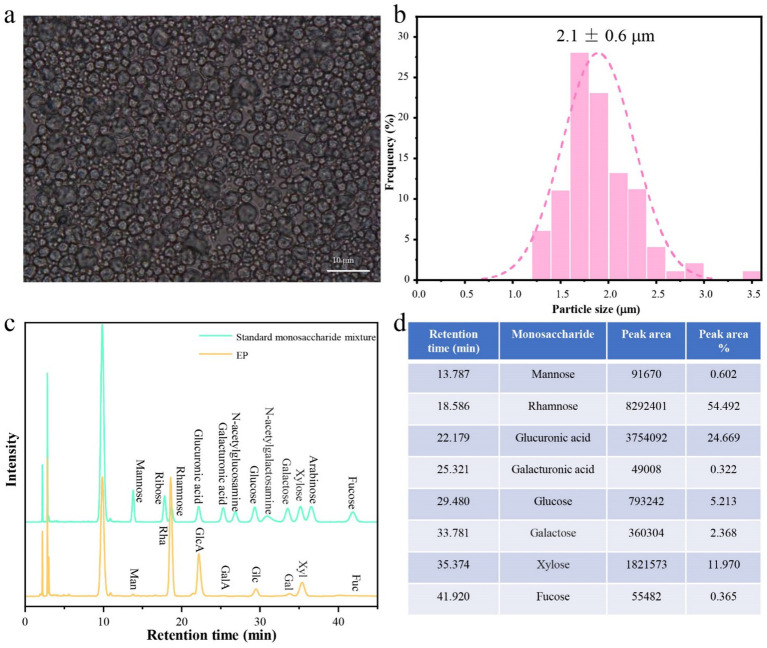
**(a)** The photograph of EDFM under optical microscope and **(b)** the statistical histogram of their particle size. **(c)** HPLC chromatogram of standard monosaccharide mixture: mannose (Man); ribose (Rib); rhamnose (Rha); Glucuronic acid (GlcA); Galacturonic acid (GalA); N-acetylglucosamine (NAG); glucose (Glc); N-acetylglucosamine; galactose (Gal); xylose (Xyl); arabinose (Ara); fucose (Fuc) and monosaccharide composition of the purified EP from EDFM. **(d)** The peak area of each peak in the HPLC curves of the purified EP from EDFM.

*Enteromorpha prolifera* used in this study contained water-soluble polysaccharide of 55.81 ± 0.30%, the crude fiber of 7.90 ± 0.30%, protein of 18.42 ± 0.08%, ash content of 9.79 ± 0.23%, crude fat of 0.36 ± 0.02% and water of 8.30 ± 0.10%, which demonstrated water-soluble polysaccharide from EP is the main chemical component. EDFM was prepared with the highest yield of 76.9 ± 4.1% by spray drying at an inlet temperature of 180°C and injection rate of 45% (Supplementary Figures S2, S3 and Supplementary Table S3). The prepared EDFM was spherical, with a relatively uniform particle size of 2.1 ± 0.6 mm (Figures 2a,b), and its chemical composition was analyzed to contain EP (73.70 ± 0.30%), protein (1.04 ± 0.04%), ash (13.71 ± 0.43%), and water (10.23 ± 0.31%). The EP purified from EDFM contained monosaccharides including mannose, rhamnose, glucuronic acid, galacturonic acid, glucose, galactose, xylose, and fucose, in a proportion of 0.602:54.492:24.669:0.322:5.213:2.368:11.970:0.365 (Figures 2c,d). Furthermore, EP was tested with a sulphate content of 6.9 ± 0.1%, and the molecular weight of EP in EDFM was 4.1 × 10^5^ Da.

### 3.2 EDFM improved general symptoms of the mice with UC

**Figure 3:**
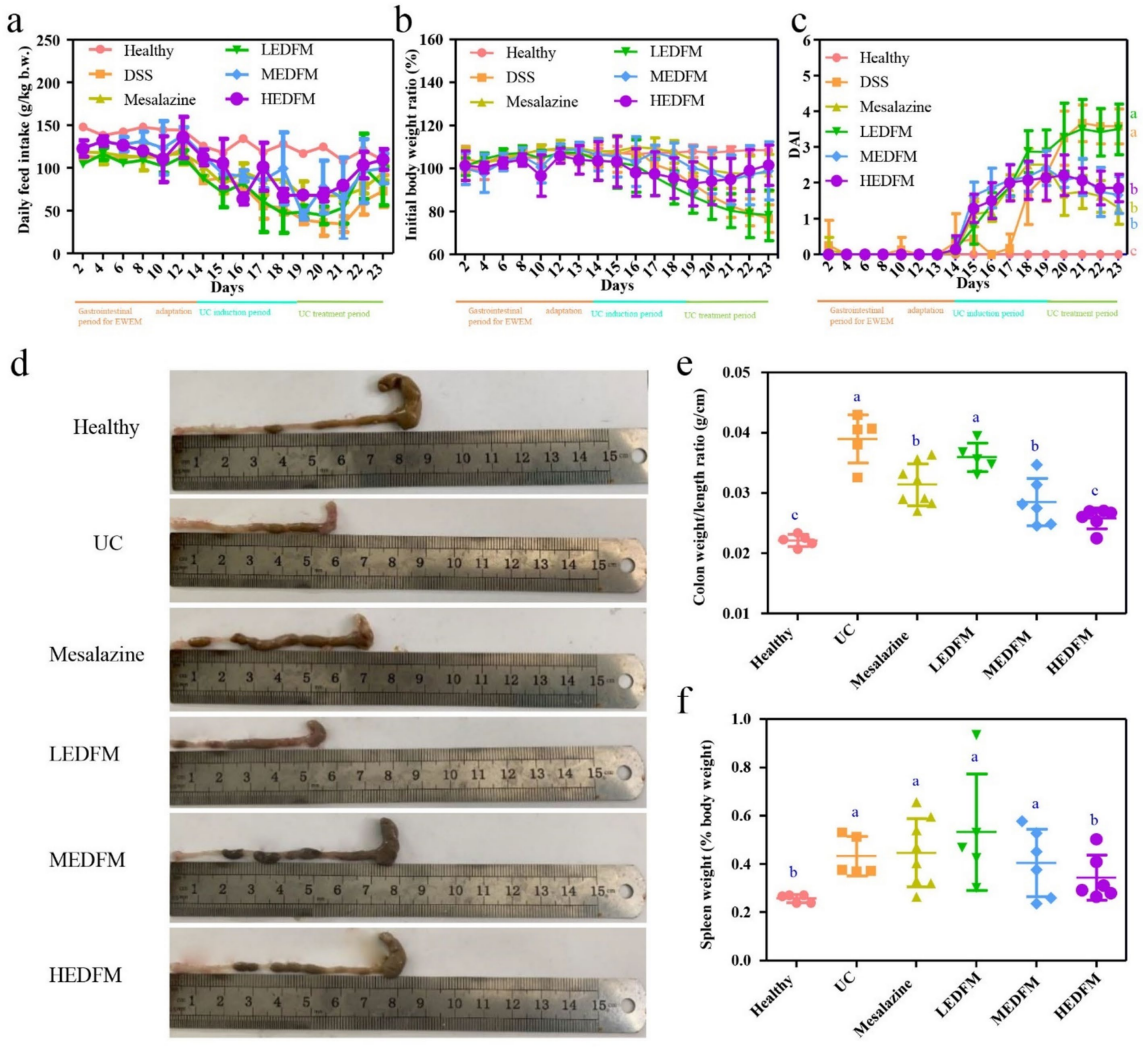
Effect of dietary supplement of EDFM on the **(a)** daily feed intake, **(b)** daily initial body weight ratio, **(c)** DAI, **(d)** colon length, **(e)** colon weight/length ratio and **(f)** spleen weight of the mice. Data are represented as the mean ± SD, *n* = 6. Data with different superscript letters indicate significant differences (*p* < 0.05).

As shown in Figures 3a,b, the mice of all experimental groups maintained a steady daily food intake, displayed a gain in body weight, and had no diarrhea in the first 14 days prior to inducing UC, suggesting the dosages of the EDFM employed in this study was within the safe limits and showed no adverse effects on the mice. After the induction of UC starting on the experiment’s 14^th^ day, the mice’s daily feed intake and body weight dramatically reduced, and their DAI scores significantly increased in all other experimental groups excluding the healthy group (Figures 3a–c). After the induction of UC stopping on the experiment’s 19^th^ day, the daily feed intake and body weight of the mice in all other experiment groups begun to rebound, and their DAI scores decreased, except for the UC and LEDFM groups (Figures 3a–c). Furthermore, the colon weight/length ratio showed that the colon weight/length ratio values of the Mesalazine, MEDFM and HEDFM groups were significantly higher than those in the UC and LEDFM groups (Figures 3d,e). However, Mesalazine, LEDFM and MEDFM treatments failed to decrease the spleen weight (% body weight), in contrast, HEDFM treatment decreased the spleen weight to the level of mice in the healthy group (Figure 3f). These results demonstrated the dietary supplement of EDFM could alleviate the general symptoms of the mice with UC at medium (38 mg EDFM /100 g) and high dosage (96 mg EDFM/100 g).

### 3.3 EDFM enhanced epithelial barrier and regulated inflammation of the mice with UC

**Figure 4:**
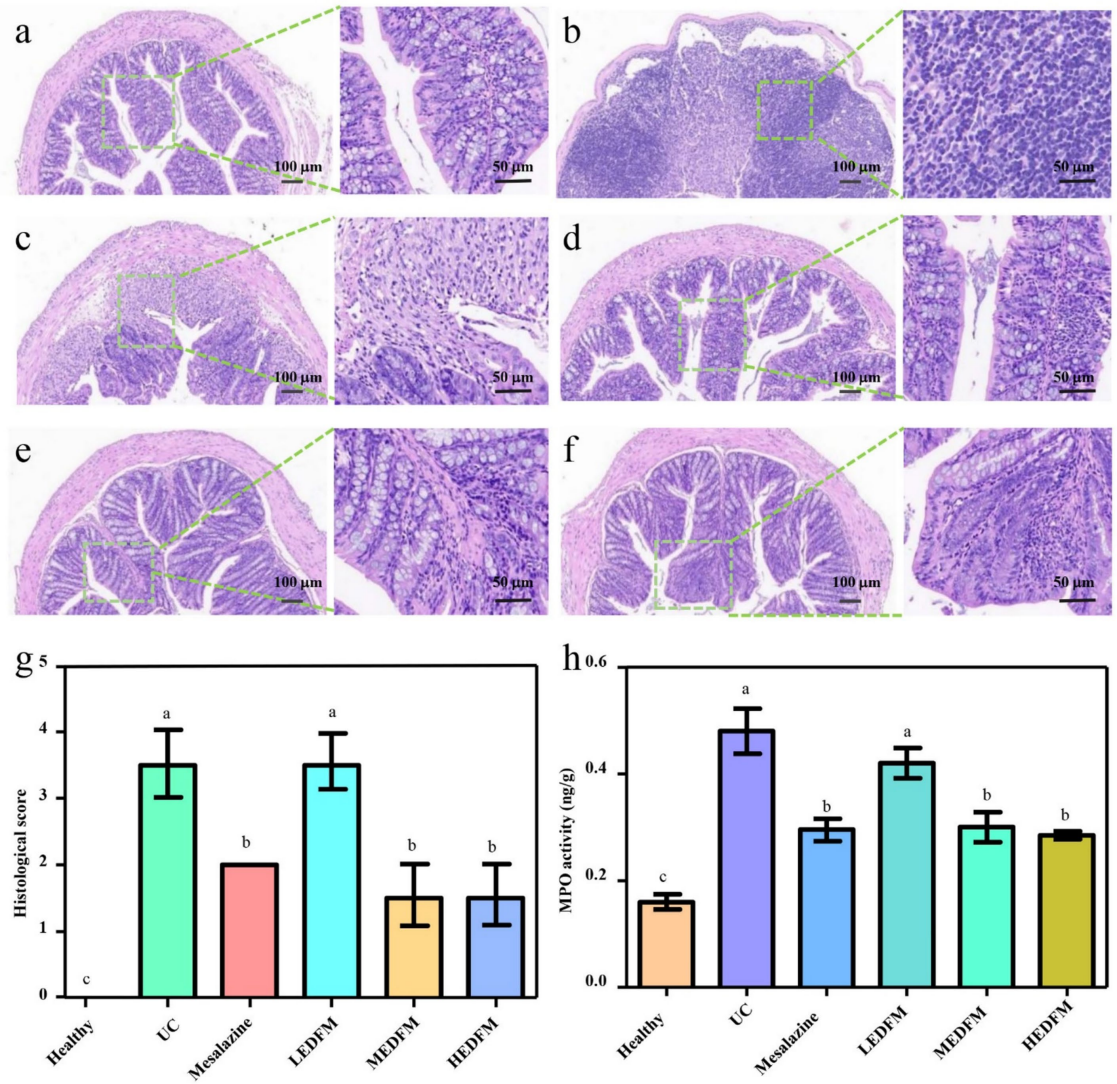
Histopathological observation of colon stained by hematoxylin and eosin. **(a)** Healthy group. **(b)** UC group. **(c)** Mesalazine group. **(d)** LEDFM group. **(e)** MEDFM group. **(F)** HEDFM group. Scale bar 100 mm and 50 mm. **(g)** Histological score and **(h)** MPO activity of the colon. Data are represented as the mean ± SD, *n* = 6. Data with different superscript letters indicate significant differences (*p* < 0.05).

The H&E images showed the colon of mice in the healthy group possessed a fully intact epithelial barrier, entire crypt structure, complete goblet cells, and no inflammatory cell infiltration (Figure 4a). Nonetheless, UC group exhibited typical characteristics of colon with abnormal structure, including the depletion of epithelial and goblet cells, crypt destruction, and substantial infiltration of inflammatory cells in the intestinal mucosa and submucosa, caused by continuous administration of DSS (Figure 4b). Except for the LEDFM group, all other experimental groups mice exhibited diminished damage, maintained a complete epithelial barrier and crypt structure, increased goblet cells, and reduced inflammatory cell infiltration (Figures 4c–f). Furthermore, the colon histological score of the mice in UC group was 3.5 ± 0.4, which was observed to be significantly higher than those observed in other groups. Figure 4g demonstrated that Mesalazine, MEDFM, and HEDFM treatment reduce the histological score to 2.0 ± 0.3, 1.5 ± 0.3, and 1.5 ± 0.4, respectively. Figure 4h illustrated a considerable downregulation of myeloperoxidase (MPO) expression in the colon of mice in Mesalazine, MEDFM and HEDFM groups, as compared to the UC group, suggesting the administration of Mesalazine, MEDFM, and HEDFM could mitigate the infiltration of inflammatory cells of the colon, as observed in H&E images.

**Figure 5:**
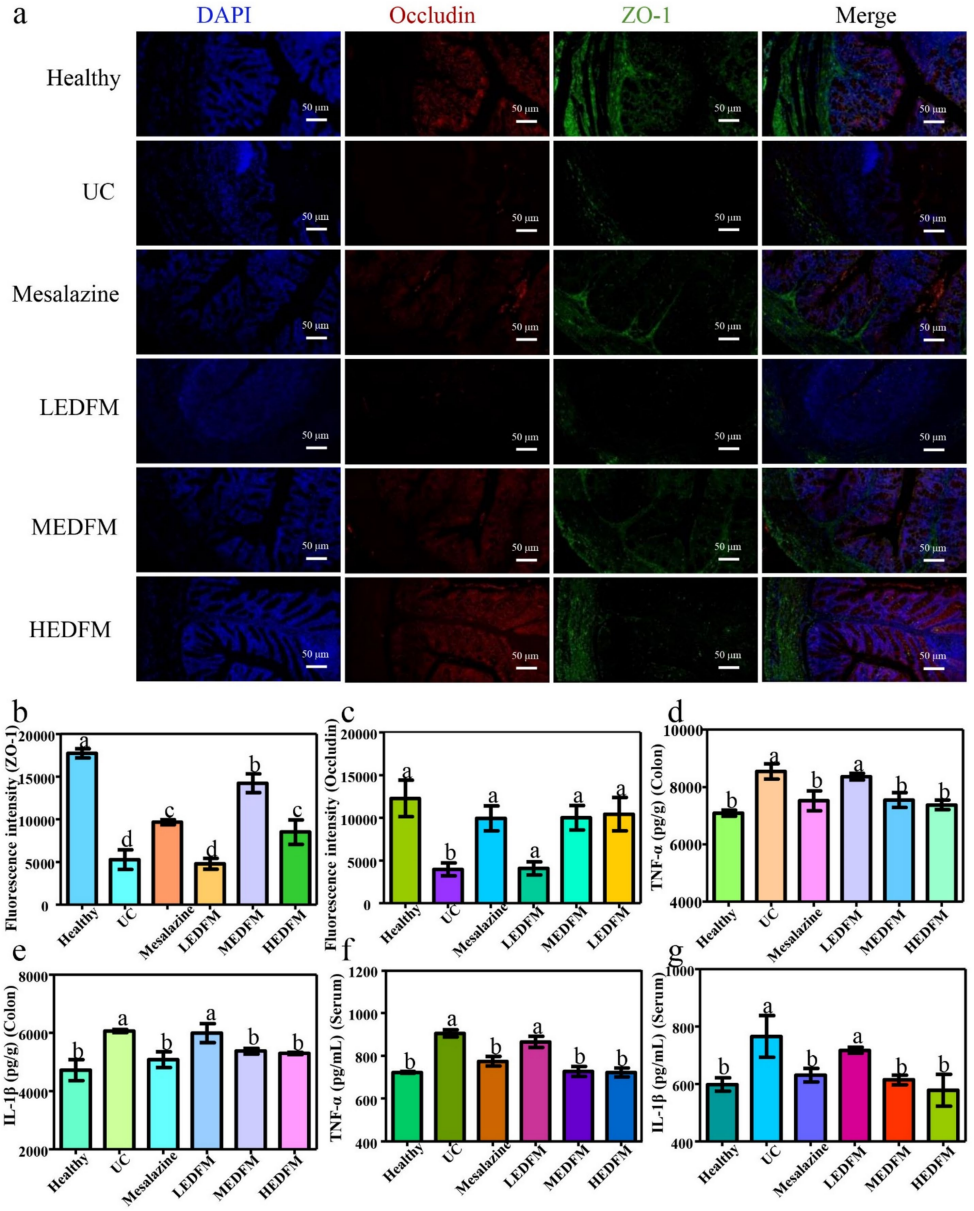
**(a)** Immunofluorescence staining of ZO-1 and occludin. Green fluorescence reflects the expression of ZO-1. Red fluorescence demonstrates the expression of occludin. Blue fluorescence represents the cell nucleus. Scale bar 50 mm. The fluorescence intensity of **(b)** ZO-1 and **(c)** occludin of the colon. The colon levels of inflammatory factors **(d)** TNF-*α* and **(e)** IL-1β. The serum levels of inflammatory factors **(f)** TNF-α and **(g)** IL-1β. Data are represented as the mean ± SD, *n* = 6. Data with different superscript letters indicate significant differences (*p* < 0.05).

As for the expression of tight junction proteins (TJs), Figures 5a–c illustrated the weak green fluorescence and red fluorescence intensity in the colon of mice in the UC group. This fluorescence intensity is approximately 30 and 32% of the fluorescence intensity observed in the colon of mice in the healthy group, respectively. However, the colon of mice in the Mesalazine, MEDFM, and HEDFM groups exhibited enhanced green and red fluorescence intensity compared to the fluorescence intensity observed in the UC group. These demonstrated the administration of Mesalazine, MEDFM, and HEDFM could maintain the integrity of the intestinal epithelial barrier by improving the expression of Occludin and ZO-1 proteins in the colon.

The expression of pro-inflammatory cytokines including tumor necrosis factor-*α* (TNF-α) and interleukin-1β (IL-1β) was evaluated later. As shown in Figures 5d–g, the levels of the expression for TNF-α and IL-1β in both the colon and serum of mice in the UC group exhibited a considerable upregulation compared to those of mice in the healthy group. This finding serves as evidence for the successful establishment of the UC model. Following the administration of Mesalazine, MEDFM, and HEDFM, a notable decrease in the levels of TNF-α and IL-1β was observed in both colon and serum, in comparison to mice in the UC group. In contrast, the supplement of LEDFM did not significantly alter TNF-α and IL-1β expression levels in the colon and serum. These findings suggested that MEDFM and HEDFM can downregulate the expression of TNF-α and IL-1β in mice with UC, leading to a mitigation of inflammatory response.

### 3.4 EDFM improved the mucus barrier of the mice with UC

**Figure 6:**
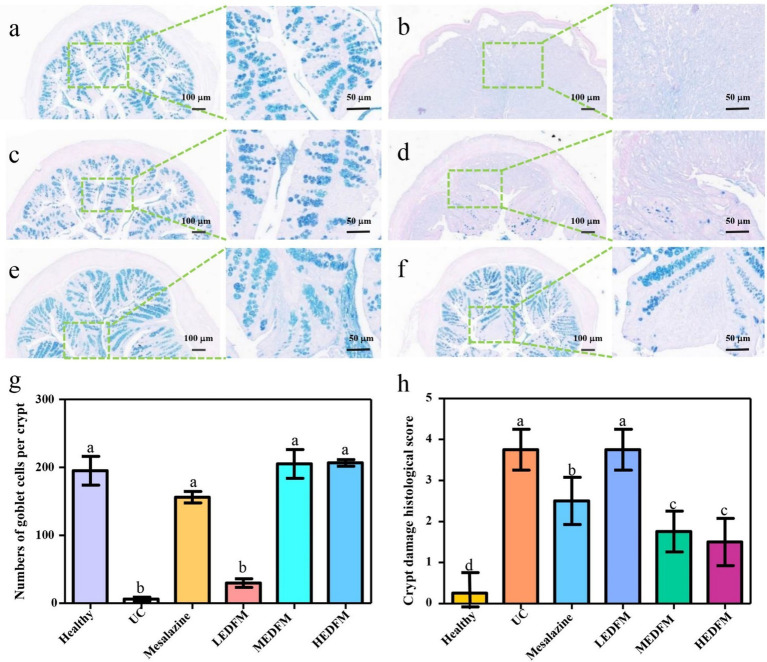
The colonic tissue sections stained by alcian blue periodic acid-Schiff. **(a)** Healthy group. **(b)** UC group. **(c)** Mesalazine group. **(d)** LEDFM group. **(e)** MEDFM group. **(f)** HEDFM group. Scale bar 100 mm and 50 mm. **(g)** The number of goblet cells per crypt of the colon. **(h)** The crypt damage histological score of the colon. Data are represented as the mean ± SD, *n* = 6. Data with different superscript letters indicate significant differences (*p* < 0.05).

Given the importance of the mucus layer in the integrity of the intestinal barrier, this study further evaluated the effect of EDFM on the mucus layer by staining the mucin in the colon with Alcian blue. Figure 6a illustrated the goblet cells located in the lamina propria were filled with mucin, and mucin was also observed on the surface of the colon epithelium of mice in the healthy group. In contrast, most of the goblet cells in the lamina propria and lumen of the colon of mice in the UC group were lost (Figures 6b,g,h), resulting in almost no mucin. Following the treatment with.

Mesalazine, MEDFM, and HEDFM, there was a notable increase in the number of goblet cells filled with mucin within each crypt located in the lamina propria of the colon. Additionally, the crypt damage score was observed to diminish, with the lamina propria becoming filled with mucin. Notably, mucin was also observed to accumulate on the surface of the colon epithelium (Figures 6c,e–h). However, there was no significance in the number of goblet cells and the crypt damage score in the colon of mice subjected to LEDFM treatment, in comparison to the UC group (Figures 6b,d,g,h). These results suggested that the treatment of Mesalazine, MEDFM, and HEDFM could effectively preserve the integrity of the mucus layer and impede the progression of UC by safeguarding the goblet cells and promoting the production and secretion of mucin.

### 3.5 EDFM modulated gut microbiota dysbiosis of the mice with UC

**Figure 7:**
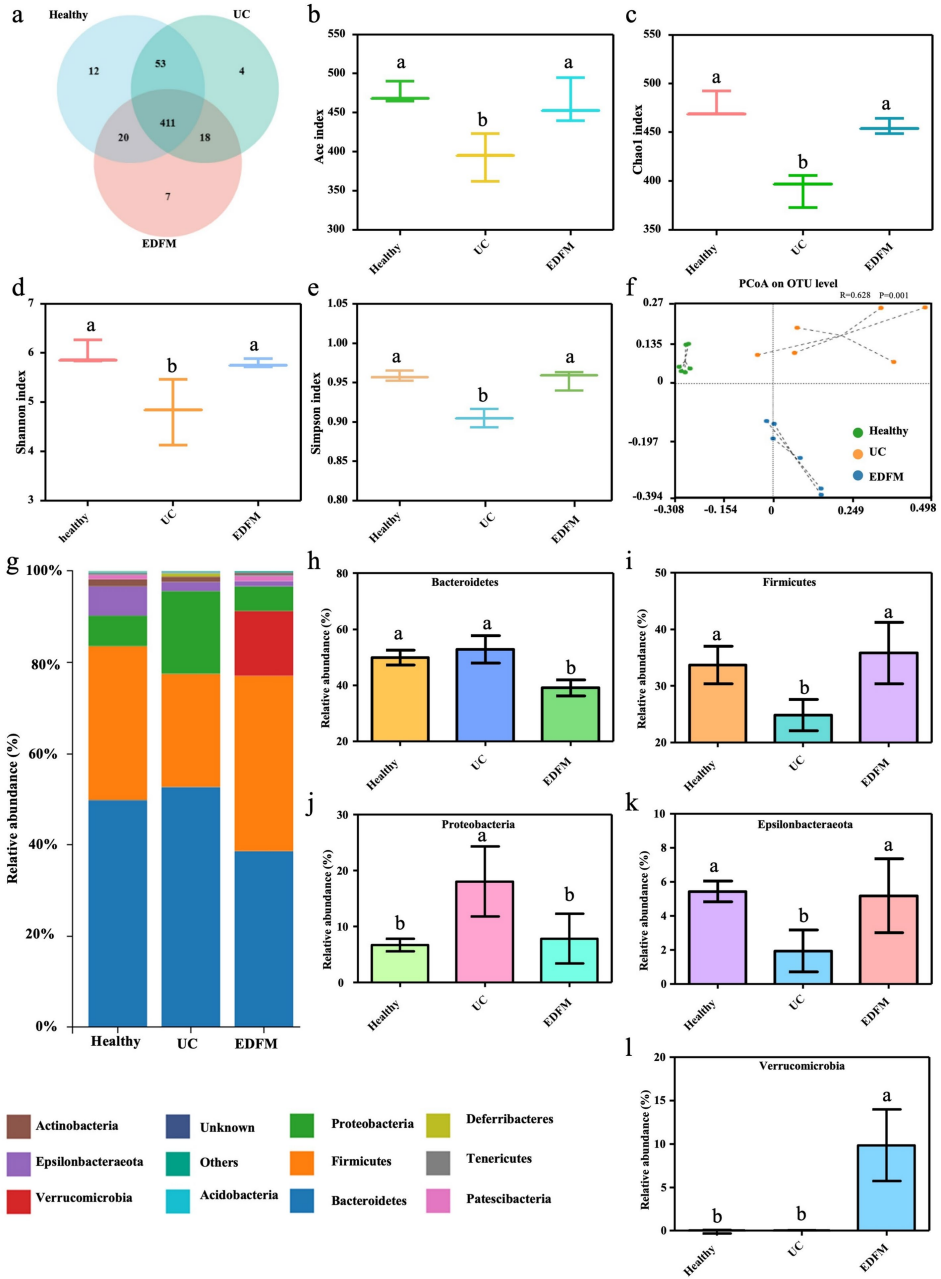
Effect of dietary supplement of EDFM on the diversity and composition of the gut microbiota at the phylum level. **(a)** Venn diagrams on the OTU level, **(b)** Ace index, **(c)** Chao1 index, **(d)** Shannon index, **(e)** Simpson index, **(f)** PCoA on the OUT level of the gut microbiota. **(g)** The Community column diagram at the phylum level and **(h–l)** the relative abundance of their top 5 phyla. Data are represented as the mean ± SD, *n* = 6. Data with different superscript letters indicate significant differences (*p* < 0.05).

To investigate whether the effect of EDFM on UC is related to the regulation of gut microbiota, 16 s rRNA gene sequencing was performed in three experimental groups (Healthy, UC, and HEDFM-treated groups) to identify alterations in the composition of the gut microbiota. The rarefaction and rank abundance curves are frequently employed in the characterization of sample diversity, indicating that sample sequencing data is reasonable and that the majority of microbial diversity in all samples has been captured (Supplementary Figures S4a,b). The Venn diagrams on the OTU level showed that 364 OTUs were shared in the feces of mice in the healthy, UC and EDFM groups. The total OTU number of mice feces in these three groups was 496, 486 and 456, respectively. The unique OTU number in the feces of mice in the healthy group (12) was a lot higher than those in the EDFM (7) and UC groups (4). These indicated that the treatment of EDFM and DSS may lead to alterations in the microbial composition (Figure 7a). Alpha diversity analysis serves as a comprehensive measure of species richness, encompassing the Chao1 and Ace indices, as well as diversity, represented by the Shannon and Simpson indices (44). Previous research shows a positive correlation between the Ace and Chao1 indices and microbial richness, and the Shannon and Simpson indices exhibit a positive correlation with microbial diversity (44). As shown in Figures 7b–e, the Ace index, Chao index, Shannon index, and Simpson index of gut microbiota of mice in the UC group exhibited a statistically significant reduction compared to those in the healthy group, suggesting a notable decline in both the diversity and richness of gut microbiota in mice with UC. The dietary supplement of EDFM resulted in a notable increase of gut microbial diversity and richness in mice, suggesting EDFM possessed the potential to mitigate the reduction in gut microbial diversity and richness observed in mice with UC effectively. For PCoA on the OTU level of *β*-diversity, the gut microbiota was distictly clustered among healthy, UC and EDFM groups and the distribution of the EDFM group was much closer to the healthy group. ANOSIM analysis showed substantial differences between the healthy, UC, and EDFM groups (*p* < 0.05). This suggested that EDFM could modulate the composition of the gut microbiota in mice, leading to a beneficial development for the body.

To facilitate a more comprehensive examination of the regulatory impact of EDFM on gut microbiota and reveal the specific composition of gut microbiota, an investigation was conducted to assess alterations in the composition and relative abundance of gut microbiota at both the phylum and genus levels. Figure 7g displayed the top 10 microbial communities at the phylum level. Notably, the relative abundance of *Bacteroidetes*, *Firmicutes*, *Proteobacteria*, *Epsilonbacteraeota*, and *Verrucomimicrobia* phyla is significantly influenced by UC. The feces of mice in the UC group showed a reduced relative abundance of *Firmicutes* and *Epsilonbacteraeota* phyla, while an increased relative abundance of *Bacteroidetes* and *Proteobacteria* phyla compared to the healthy group (Figures 7h–l). Compared with the mice in the UC group, the dietary supplement of EDFM resulted in a notable rise in the relative abundance of *Firmicutes* and *Verrucomimicrobia* phyla within the gut microbiota of mice. Conversely, the abundance of *Bacteroidetes* and *Proteobacteria* phyla exhibited a drop.

**Figure 8:**
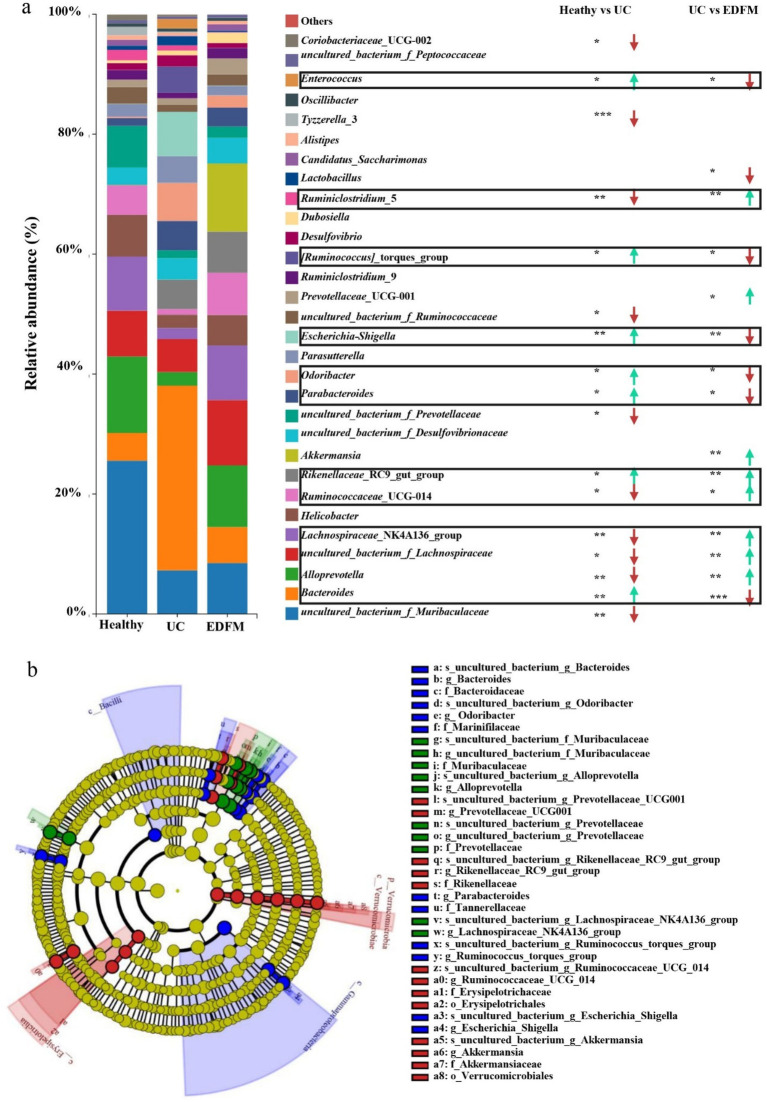
Effect of dietary supplement of EDFM on the gut microbiota. **(a)** The column diagram of the top 30 genera at the genus level. The statistically significant difference was analyzed by the Wilcoxon rank-sum test and Kruskal−Wallis H test. *p < 0.05, **p < 0.01, and ***p < 0.001, *n* = 6 (* represents Healthy/EDFM vs. UC). **(b)** Differentially abundant microbial cladogram obtained by LEfSe.

Furthermore, the top 30 microbial communities at the genus level were shown in Figure 8a. Among these 30 genera, seven were upregulated, and ten were downregulated in the feces of mice in the UC group compared to the healthy group. Nevertheless, the dietary supplement of EDFM successfully mitigated these detrimental alterations, resulting in a reduction in the relative abundance of pathogenic bacteria, including *Bacteroides, Escherichia-Shigella* and *Enterococcus*, and an increase in the relative abundance of probiotics, specifically *Alloprevotella, Lachnospiraceae_*NK4A136_group*, uncultured_bacterium_f_Lachnospiraceae, Ruminococcaceae_*UCG-014 and *Ruminiclostridium_*5. In addition to the upregulation of pathogenic bacteria, probiotics in the gut microbiota of mice with UC are downregulated, such as *Muribaculaceae*, *Rikenellaceae*, *Lachnospiraceae*, *Ruminococcaceae*, and *Prevotellaceae.* Here, EDFM successfully downregulated the relative abundance of *Bacteroides, Escherichia-Shigella, Enterococcus* and upregulated the relative abundance of *Alloprevotella*, *uncultured_bacterium_f_Lachnospiraceae*, *Lachnospiraceae_*NK4A136_group, *Ruminococcaceae_*UCG-014, *Ruminiclostridium_*5 in gut microbiota of mice with UC. Notably, compared to the mice in the healthy and UC groups, *Akkermansia* was substantially upregulated in the EDFM group.

To further investigate the differences in microbial communities between groups, we searched for species with significant differences in abundance between groups (i.e., biomarkers) and identified the dominant microorganisms in each group using LEfSe linear discriminant analysis (LDA). This analysis first employs the nonparametric Kruskal-Wallis rank sum to identify species whose abundances differ significantly between groups. Then, the Wilcoxon rank sum is used to test the consistency of differences between subgroups of distinct species determined in the preceding phase. Finally, linear regression analysis (LDA) is utilized to estimate the extent of each species’s impact on the difference effect. Supplementary Figure S4c illustrated the distribution of LDA values for species with differences between groups, highlighting species with significant differences in abundance under conditions where the LDA score was greater than 4. As shown in Supplementary Figure S4c, there were 10, 16, and 14 significantly different taxonomic groups at different classification levels in the Healthy, UC, and EDFM groups, respectively. The evolutionary branching diagrams of these species with significantly different taxonomic groups were shown in Figure 8b. It could be seen from Figure 8b that the dominant bacteria in each group were different and that the dominant bacteria in the healthy group were f_*Muribaculaceae*, g*_Alloprevotella*, f_*Prevotellaceae*, g_*Lachnospiraceae_*NK4A136_group, while f_*Bacteroidaceae*, f_*Marinifilaceae*, g_*Odoribacter*, f_*Tannerellaceae*, g_*Parabacteroides*, g_*Ruminococcus_torques_group*, g_*Escherichia_Shigella* in the UC group and g_*Prevotellaceae_*UCG001, g_*Rikenellaceae_*RC9_gut_group, f_*Rikenellaceae*, g_*Ruminococcaceae_*UCG_014, o_*Erysipelotrichales*, g_*Akkermansia* in the EDFM group. These could be biomarkers for the diagnosis and prognosis of UC.

**Figure 9:**
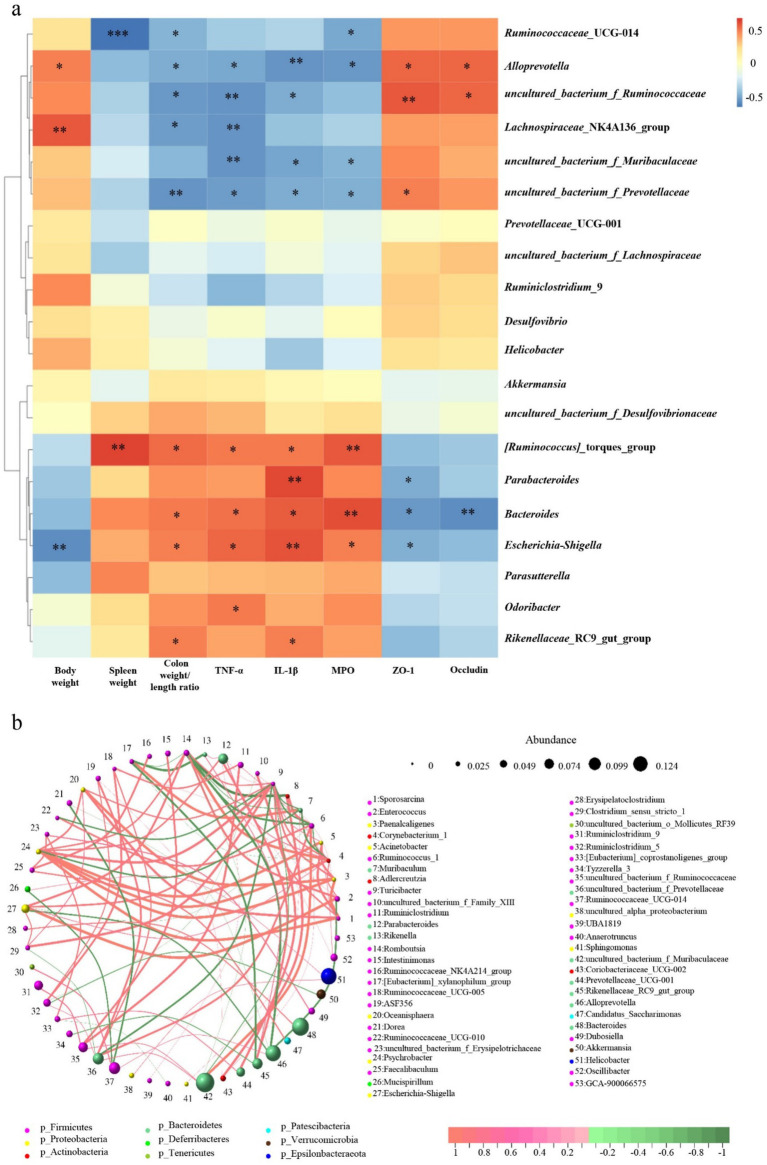
**(a)** Heat map representation of Spearman rank correlation between different gut microbiota and UC-related indicators. *p < 0.05, **p < 0.01, and ***p < 0.001, *n* = 6. **(b)** Circle layout correlation network of gut microbiota.

Furthermore, Spearman rank correlation analysis was employed to examine the potential correlations between significantly altered taxa in the gut microbiota and UC-related indicators. As shown in Figure 9a, 12 genera were correlated with at least one UC-related indicator negatively or positively (*p* < 0.05, marked as *). Among them, *[Ruminococcus]_ Torques_ Group*, *Parabacteroides*, *Bacteroides*, *Escherichia*-*Shigella* were positively correlated with spleen weight, colon weight/length ratio, TNF-*α*, IL-1β and MPO, indicating they were related with the development of UC, while *Ruminococcaceae_*UCG-014*, Alloprevotella, uncultured_bacterium_f_Ruminococcaceae, Lachnospiraceae_*NK4A136_group*, uncultured_bacterium_f_Muribaculaceae, uncultured_bacterium_f_Prevotellaceae* were negatively correlated with them, indicating they were relative with the alleviation of UC. Besides, *Alloprevotella, uncultured_bacterium_f_Ruminococcaceae* and *uncultured_bacterium_f_Prevotellaceae* were positively correlated with ZO-1 and Occludin, indicating they were related to the destruction of the integrity of the intestinal barrier. At the same time, *Parabacteroides*, *Bacteroides and Escherichia-Shigella* were negative with them, demonstrating they were related to the repair of intestinal barrier integrity.

Moreover, a circle layout correlation network analysis was performed on the microbial community to investigate the association between distinct microbial communities. As depicted in Figure 9b, a densely interconnected network was observed, suggesting the presence of various microbial communities that exert either facilitative or inhibitory impacts. The correlation between the genera of bacteria associated with UC was analyzed. The *Ruminococcaceae_*UCG-014 and *Alloprevotella* genera were connected by red lines, indicating a positive correlation between the two genera. The *Alloprevotella* and *Escherichia-Shigella* genera were connected by green lines, showing a negative correlation between the two genera. In addition, the *Uncultured_ Bacterium_ F_ Ruminococcaceae* genus and *uncultured_ Bacterium_ F_ Muribaculaceae* genus showed a positive correlation. *Uncultured_ Bacterium_ F_ Prevotelaceae* genus and *uncultured_ Bacterium_ F_ Muribaculaceae* genus showed a positive correlation. *Parabacteroides* genus and *Rikenellaceae_*RC9_gut_group genus showed a positive correlation. These results indicated a mutually promoting effect among the genera associated with the alleviation of UC and among those related to the development of UC. In contrast, the genera associated with the alleviation of UC and those associated with UC development had a direct or indirect inhibitory effect.

## 4 Discussion

Dietary fiber, categorized into soluble and insoluble types, is known for its health-promoting effects, which vary depending on its source, structure, and composition (16). Among marine seaweeds, *E. prolifera* is a type of edible green algae rich in soluble dietary fiber, which includes water-soluble polysaccharides, crude fiber, protein, minerals, fats, etc. The main chemical component of *E. prolifera* used in this study was water-soluble polysaccharide (EP), whose main monosaccharides composition was rhamnose and glucuronic acid (Figures 2c-d). This chemical structure could confer EP excellent antioxidant capability (45–47). Additionally, glucose, galactose and xylose in EP could stimulated the growth profile of some probiotic bacteria and higher production of short-chain fatty acids (SCFAs) (48, 49). Furthermore, EP had a sulphate content of 6.9 ± 0.1%, which indicated that EP was sulfated polysaccharide. Compared with non-sulfated polysaccharide, sulfated polysaccharide showed stronger (*p* < 0.05) anti-inflammatory activity (50). In addition, the molecular weight of EP in EDFM was 4.1 × 10^5^ Da. Previous studies have demonstrated the antioxidant and immunomodulatory activities of EP (6 × 10^5^ Da, with a sulfate content of 11.5%) (51). These properties suggest that EDFM has significant potential to treat UC through its antioxidant, anti-inflammatory, and gut microbiota-modulating abilities. Specifically, the sulfated rhamnose structure of EP may enhance its ability to interact with intestinal epithelial cells and modulate immune responses, while its monosaccharide of glucose, galactose and xylose promotes the growth of beneficial bacteria such as *Alloprevotella* and *Lachnospiraceae* and the production of SCFAs (44, 45).

EDFM dietary supplement could improve typical clinical symptoms of colitis which was evidence by the reduction of DAI values, colon and spleen index (Figure 3), suggesting its potential therapeutic efficacy in UC (52). The improvement of the above clinical parameters is likely due to its anti-inflammatory and antioxidant abilities, thus maintaining the integrity of intestinal barrier (53). During the development of UC, the colon inflammation gradually exacerbates, characterized by a significant infiltration of inflammatory cells. This infiltration ultimately results in the impairment of the colon’s epithelial function and the dysregulation of TJs (54). The administration of Mesalazine, MEDFM, and HEDFM could mitigate the infiltration of inflammatory cells of the colon, as observed in H&E images (Figure 4c,e,f). EDFM also downregulates the expression of MPO (Figure 4h), which is served as an indicator for the recruitment of neutrophils (55), hence providing additional confirmation that it can mitigate the infiltration of inflammatory cells of the colon. Meanwhile, our study showed that the administration of MEDFM and HEDFM could both improve the expression of Occludin and ZO-1 proteins in the colon (Figures 5a–c), which are fundamental constituents of the intestinal epithelial barrier (56). This suggests that EDFM supplementation helps maintain the integrity of the intestinal epithelial barrier, which can be attributed to the presence of EP in EDFM. Previous studies have showed EP can enhanced the gut barrier integrity of obese mice (21). However, the specific regulatory mechanism and receptor related to TJs protein expression promoted by EP need further explored.

Furthermore, MEDFM and HEDFM can downregulate the expression of pro-inflammatory cytokines including TNF-*α* and IL-1β in mice with UC (Figures 5d–g), leading to a mitigation of inflammatory response (57). Our previous research have demonstrated that EP can downregulate the expression of IL-1β, IL-6, and TNF-α through TLR2-dependent NF-κB, PKC/ERK/MAPK, and PI3K/Akt signaling pathways (51). This suggests that the anti-inflammatory effects of MEDFM and HEDFM in UC may be attributed to the regulatory role of EP in these signaling pathways. Thereby the above findings confirm that EDFM supplementation plays a crucial role in mitigating inflammatory response.

Moreover, the treatment of MEDFM and HEDFM effectively protected goblet cells and promoted mucin production (Figure 6). This effect may be attributed to the fermentation of EDFM in the colon, which enhances the production of short-chain fatty acids, known to stimulate mucin production (58). The mucus layer, a key component of the intestinal barrier, serves as the first line of defense, preventing direct interactions between pathogens, toxins, and the intestinal epithelium (41). Additionally, it creates a favorable environment and provides nourishment for beneficial bacteria (19). Therefore, EDFM supplementation helps preserve the integrity of the mucus layer, contributing to the protection and maintenance of the intestinal barrier and potentially impeding the progression of UC.

Additionally, the gut microbiota is a critical component in the maintenance of intestinal homeostasis, and the dysregulation of gut microbiota is a significant contributing factor in the pathogenesis of UC (59). In our study, we found a notable decline in both the diversity and richness of gut microbiota in mice with UC (Figures 7b–e), which is consistent with other research showing that UC leads to disturbances in the gut microbiota, resulting in reduced microbial diversity and richness (30). Interestingly, EDFM dietary treament effectively improved the diversity and richness (Figures 7b–e), confirming its potential to regulate the gut microbiota. In addition, UC mice exhibited dereased abundance of *Firmicutes* phylum (42) and increased abundance of *Proteobacteria_Enterobacteriaceae* and *Bacteroidota_Bacteroides* (60), and EDFM dietary supplement regulate it to normal level (Figures 7h–l). Consistent with our findings, a number of studies have reported poor microbial diversity, unchecked expansion of *Proteobacteria* and *Bacteroidetes* phyla, and depletion of *Firmicutes* phylum in UC patients (57, 61). Thus, the above results preliminarily suggest that EDFM supplementation may help restore gut microbiota homeostasis and potentially offer therapeutic benefits for UC.

The analysis of gut microbiota composition at the genus level provides further evidence. Specifically, the relative abundance of pathogenic bacteria, including *Bacteroides*, *Escherichia-Shigella*, and *Enterococcus*, was significantly increased in UC mice, while beneficial bacteria such as *Muribaculaceae*, *Rikenellaceae, Lachnospiraceae*, *Ruminococcaceae*, and *Prevotellaceae*, which are associated with fiber degradation and SCFAs production (62, 63), were markedly reduced (Figure 8). However, dietary supplementation with EDFM effectively reversed these dysbiotic changes by suppressing harmful bacteria while enriching probiotics such as *Alloprevotella*, *Lachnospiraceae_NK4A136_group, uncultured_bacterium_f_Lachnospiraceae, Ruminococcaceae_UCG-014,* and *Ruminiclostridium_5* (Figure 8), indicating its potential to restore microbial homeostasis. The beneficial effects of EDFM on gut microbiota may be attributed to its high EP content, which facilitates microbiota modulation. The dysbiosis observed in UC is characterized by an overabundance of pathogenic bacteria that exacerbate intestinal inflammation. For example, *Escherichia-Shigella*, a member of *Proteobacteria* phylum, is a gram-negative bacterium with an outer membrane rich in lipopolysaccharides (LPS), also known as endotoxins. LPS can activate pattern recognition receptors such as Toll-like receptor 4 (TLR4), triggering the MAPK/NF-κB signaling pathway and leading to the production of pro-inflammatory cytokines like TNF-*α*, thereby exacerbating intestinal inflammation (64). Additionally, *Enterococcus* strains isolated from UC patients have been shown to induce colitis in genetically susceptible mice (65, 66), while excessive *Bacteroides* in the gut microbiota can negatively impact gut immune function (67). Numerous studies highlight the gut microbiota-modulating effects of polysaccharides, promoting intestinal homeostasis and alleviating UC. For instance, inulin increases the abundance of probiotics such as *Muriaculaceae* (S24-7), *Prevotellaceae*, and *Rikenellaceae,* mitigating UC in mice (68). Similarly, *Gastrodia elata* polysaccharides and arabinogalactan from *Lycium barbarum* can enhance *Ligilactobacillus, Alloprevotella, Lactospiraceae* and *Ruminococcaceae* (42, 63). Notably, our results showed a significant upregulation of *Akkermansia* in the EDFM group compared to both the UC and healthy groups. *Akkermansia,* a gram-negative anaerobe belonging to *Verrucomicrobia*, is known to reside at the outermost layer of the gastrointestinal tract, where it utilizes mucin as its primary energy source (69). Despite consuming mucin, it paradoxically stimulates mucin (MUC2) expression, enhancing mucus secretion and intestinal barrier integrity (70). Numerous studies have demonstrated a negative correlation between *Akkermansia* and metabolic disorders in hosts, including enteritis, obesity, and diabetes (71, 72). Hence, we propose that EDFM supplementation may enhance *Akkermansia* abundance, promoting mucus synthesis and reinforcing the intestinal barrier.

By analyzing the correlation between UC-related indicators (Body weight, Spleen weight, Colon weight/length ratio, TNF-α, IL-1β, MPO, ZO-1, Occludin) and significantly altered taxa in the gut microbiota, key microbial populations and its possible function in the development and treatment of UC can be identified. Spearman rank correlation analysis revealed *Alloprevotella*, *uncultured_bacterium_f_Ruminococcaceae, Ruminococcaceae_UCG-014*, *Lachnospiraceae_NK4A136_group* were positively correlated with improved intestinal barrier function and alleviated inflammation; whereas *Parabacteroides*, *Bacteroides*, *Escherichia-Shigella Rikenellaceae_RC9_gut_group, Odoribacter*, and *[Ruminococcus]_torques_group* were positively associated with intestinal barrier disruption, aggravated inflammation, and worsening clinical symptoms of UC (Figure 9). Combining the results of alteration in microbial abundance (Figure 8), we think the therapeutic effect of EDFM on UC may be achieved by promoting the proliferation of UC remission-related microbial populations (*Alloprevotella*, *Ruminococcaceae_UCG-014*, *Lachnospiraceae_NK4A136_group*) while inhibiting the proliferation of UC development-related microbial populations (*Escherichia-Shigella, Parabacteroides*, *Rikenellaceae_RC9_gut_group, Odoribacter*, *[Ruminococcus]_torques_group*), thereby maintaining intestinal microbiota homeostasis and ultimately improving the pathological state of UC. However, correlation does not imply causation, and therefore, it is necessary to integrate multi-omics technologies, such as metabolomics and transcriptomics, to systematically validate the specific roles of these microbial populations in the pathogenesis of UC.

**Figure 10:**
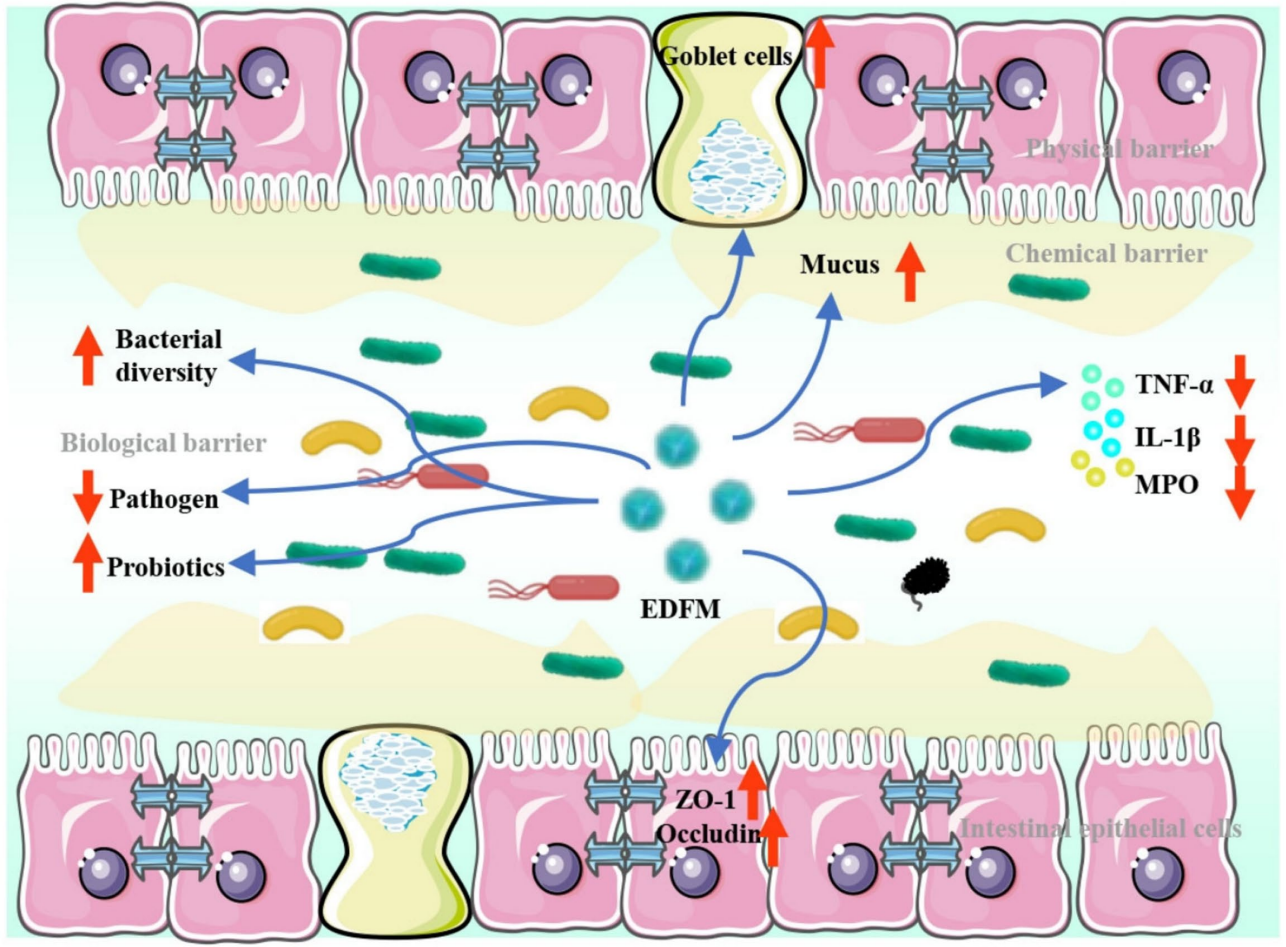
The effective mechanism underlying EDFM treatment for UC.

In conclusion, the dietary supplement of EDFM has been found to mitigate inflammation of the colon, as well as a decrease levels of pro-inflammatory cytokines. Furthermore, it could upregulate the expression of ZO-1 and occludin, thereby maintaining the integrity of the intestinal mechanical barrier. Additionally, it can increase the number of goblet cells and stimulate the synthesis and secretion of mucus, thereby facilitating the restoration of the intestinal chemical barrier. In addition, it could augment the diversity and abundance of the gut microbiota, stimulate the proliferation of microbes that produce short-chain fatty acids, and inhibit the growth of pathogenic microbes, thereby reinstating the integrity of the intestinal biological barrier (Figure 10). Our research offers a dietary approach for treating UC by repairing the integrity of the intestinal barrier, presenting a potential alternative or supplementary dietary intervention for UC prevention and treatment. However, additional research is required to elucidate the intricate mechanism by which it treats UC prior to its use.

## Data Availability

The original contributions presented in the study are included in the article/Supplementary material, further inquiries can be directed to the corresponding author.

## References

[1] Wang M Cha R Hao W Jiang X. Nanocrystalline cellulose modulates dysregulated intestinal barriers in ulcerative colitis. ACS Nano. (2023) 17:18965–78. doi: 10.1021/acsnano.3c04569, PMID: 37747898

[2] Kaplan GG. The global burden of IBD: from 2015 to 2025. Nat Rev Gastroenterol Hepatol. (2015) 12:720–7. doi: 10.1038/nrgastro.2015.150, PMID: 26323879

[3] Ng SC Shi HY Hamidi N Underwood FE Tang W Benchimol EI. The worldwide incidence and prevalence of inflammatory bowel disease in the 21st century: a systematic review of population-based studies. Gastroenterology. (2017) 390:2769–78. doi: 10.1016/S0140-6736(17)32448-0, PMID: 29050646

[4] Qian K Chen S Wang J Sheng K Wang Y Zhang M. A β-N-acetylhexosaminidase Amuc_2109 from *Akkermansia muciniphila* protects against dextran sulfate sodium-induced colitis in mice by enhancing intestinal barrier and modulating gut microbiota. Food Funct. (2022) 13:2216–27. doi: 10.1039/D1FO04094D 35133390

[5] Olén O Erichsen R Sachs MC Pedersen L Halfvarson J Askling J. Colorectal cancer in ulcerative colitis: a Scandinavian population-based cohort study. Lancet. (2020) 395:123–31. doi: 10.1016/S0140-6736(19)32545-0, PMID: 31929014

[6] Ordás I Eckmann L Talamini M Baumgart DC Sandborn WJ. Ulcerative colitis. Lancet. (2012) 380:1606–19. doi: 10.1016/S0140-6736(12)60150-0 22914296

[7] Cao Y Gao J Zhang L Qin N Zhu B Xia X. Jellyfish skin polysaccharides enhance intestinal barrier function and modulate the gut microbiota in mice with DSS-induced colitis. Food Funct. (2021) 12:10121–35. doi: 10.1039/D1FO02001C, PMID: 34528649

[8] Kuo W-T Zuo L Odenwald MA Madha S Singh G Gurniak CB. The tight junction protein ZO-1 is dispensable for barrier function but critical for effective mucosal repair. Gastroenterology. (2021) 161:1924–39. doi: 10.1053/j.gastro.2021.08.047, PMID: 34478742 PMC8605999

[9] Liu Y Yu X Zhao J Zhang H Zhai Q Chen W. The role of MUC2 mucin in intestinal homeostasis and the impact of dietary components on MUC2 expression. Int J Biol Macromol. (2020) 164:884–91. doi: 10.1016/j.ijbiomac.2020.07.191, PMID: 32707285

[10] Bekkers M Stojkovic B Kaiko GE. Mining the microbiome and microbiota-derived molecules in inflammatory bowel disease. Int J Mol Sci. (2021) 22:11243. doi: 10.3390/ijms222011243, PMID: 34681902 PMC8540913

[11] Li J Hou W Lin S Wang L Pan C Wu F. Polydopamine nanoparticle-mediated dopaminergic immunoregulation in colitis. Adv Sci. (2022) 105:1544–53. doi: 10.1002/advs.202104006 PMC8728836 34713621

[12] Hao W Cha R Wang M Zhang P Jiang X. Impact of nanomaterials on the intestinal mucosal barrier and its application in treating intestinal diseases. Nanoscale Horiz. (2022) 7:6–30. doi: 10.1039/D1NH00315A, PMID: 34889349

[13] Zhu W Winter MG Byndloss MX Spiga L Duerkop BA Hughes ER. Precision editing of the gut microbiota ameliorates colitis. Nature. (2018) 553:208–11. doi: 10.1038/nature25172, PMID: 29323293 PMC5804340

[14] Bach Knudsen KE Lærke HN Hedemann MS Nielsen TS Ingerslev AK Gundelund Nielsen DS. Impact of diet-modulated butyrate production on intestinal barrier function and inflammation. Nutrients. (2018) 10:1499. doi: 10.3390/nu10101499, PMID: 30322146 PMC6213552

[15] Yuan D Li C Huang Q Fu X Dong H. Current advances in the anti-inflammatory effects and mechanisms of natural polysaccharides. Crit Rev Food Sci Nutr. (2022) 63:5890–910. doi: 10.1080/10408398.2022.2025535, PMID: 35021901

[16] Maurer LH Cazarin CB Quatrin A Minuzzi NM Costa EL Morari J. Grape peel powder promotes intestinal barrier homeostasis in acute TNBS-colitis: a major role for dietary fiber and fiber-bound polyphenols. Food Res Int. (2019) 123:425–39. doi: 10.1016/j.foodres.2019.04.068, PMID: 31284994

[17] Praveen MA Parvathy KRK Balasubramanian P Jayabalan R. An overview of extraction and purification techniques of seaweed dietary fibers for immunomodulation on gut microbiota. Trends Food Sci Tech. (2019) 92:46–64. doi: 10.1016/j.tifs.2019.08.011

[18] Thomas H. Low dietary fibre induces colonic mucus layer erosion by microbiota. Nat Rev Gastroenterol Hepatol. (2017) 14:4. doi: 10.1038/nrgastro.2016.197, PMID: 27924078

[19] Desai M Seekatz A Koropatkin N Kamada N Martens E. A dietary fiber-deprived gut microbiota degrades the colonic mucus barrier and enhances pathogen susceptibility. Cell. (2016) 167:1339–53.e21. doi: 10.1016/j.cell.2016.10.043, PMID: 27863247 PMC5131798

[20] Wu W Hu J Gao H Chen H Fang X Mu H. The potential cholesterol-lowering and prebiotic effects of bamboo shoot dietary fibers and their structural characteristics. Food Chem. (2020) 332:127372. doi: 10.1016/j.foodchem.2020.127372, PMID: 32615381

[21] Zou T Xie F Liang P Chen J Wang Z Du M. Polysaccharide-rich fractions from *Enteromorpha prolifera* improve hepatic steatosis and gut barrier integrity in high-fat diet-induced obese mice linking to modulation of gut microbiota. Biomed Pharmacother. (2023) 157:114034. doi: 10.1016/j.biopha.2022.114034, PMID: 36434956

[22] Wassie T Niu K Xie C Wang H Xin W. Extraction techniques, biological activities and health benefits of marine algae *Enteromorpha prolifera* polysaccharide. Front Nutr. (2021) 8:747928. doi: 10.3389/fnut.2021.747928, PMID: 34692752 PMC8529069

[23] Li J Jiang F Chi Z Han D Yu L Liu C. Development of *Enteromorpha prolifera* polysaccharide-based nanoparticles for delivery of curcumin to cancer cells. Int J Biol Macromol. (2018) 112:413–21. doi: 10.1016/j.ijbiomac.2018.02.002, PMID: 29410267

[24] Zhou Z Pan S Wu S. Modulation of the growth performance, body composition and nonspecific immunity of crucian carp *Carassius auratus* upon *Enteromorpha prolifera* polysaccharide. Int J Biol Macromol. (2020) 147:29–33. doi: 10.1016/j.ijbiomac.2020.01.065, PMID: 31923485

[25] Cui Y Zhu L Li Y Jiang S Sun Q Xie E. Structure of a laminarin-type β-(1→3)-glucan from brown algae *Sargassum henslowianum* and its potential on regulating gut microbiota. Carbohyd Polym. (2021) 255:117389. doi: 10.1016/j.carbpol.2020.117389, PMID: 33436218

[26] Xu Y Li J Han X Zhang Z Zhong M Hu Z. *Enteromorpha prolifera* diet drives intestinal microbiome composition in *Siganus oramin*. Curr Microbiol. (2020) 78:229–37. doi: 10.1007/s00284-020-02218-6 33034768

[27] Yuan X Zheng J Ren L Jiao S Feng C du Y. *Enteromorpha prolifera* oligomers relieve pancreatic injury in streptozotocin (STZ)-induced diabetic mice. Carbohydr Polym. (2019) 206:403–11. doi: 10.1016/j.carbpol.2018.11.019, PMID: 30553339

[28] Zhao A Chen Y Li Y Lin D Yang Z Wang Q. Sulfated polysaccharides from *Enteromorpha prolifera* attenuate lipid metabolism disorders in mice with high-fat diet-induced obesity via an AMPK-dependent pathway. J Nutr. (2021) 152:939–49. doi: 10.1093/jn/nxab432, PMID: 34958377

[29] Guo F Han M Lin S Ye H Chen J Zhu H. *Enteromorpha prolifera* polysaccharide prevents high-fat diet-induced obesity in hamsters: a NMR-based metabolomic evaluation. J Food Sci. (2021) 86:3672–85. doi: 10.1111/1750-3841.15818, PMID: 34191277

[30] Liu X Zhang Y Li W Zhang B Yin J Liu Q. Fucoidan ameliorated dextran sulfate sodium-induced ulcerative colitis by modulating gut microbiota and bile acid metabolism. J Agr Food Chem. (2022) 70:14864–76. doi: 10.1021/acs.jafc.2c06417, PMID: 36378195

[31] Fernandez-Tome S Hernandez-Ledesma B Chaparro M Indiano-Romacho P Bernardo D Gisbert J. Role of food proteins and bioactive peptides in inflammatory bowel disease. Trends Food Sci Technol. (2019) 88:194–206. doi: 10.1016/j.tifs.2019.03.017

[32] Van Der Schoot A Creedon A Whelan K Dimidi E. The effect of food, vitamin, or mineral supplements on chronic constipation in adults: a systematic review and meta-analysis of randomized controlled trials. Neurogastroenterol Motil. (2023) 35:e14613. doi: 10.1111/nmo.14613, PMID: 37243443

[33] Premarathna AD Tuvikene R Fernando PHP Adhikari R Perera MCN Ranahewa TH. Comparative analysis of proximate compositions, mineral and functional chemical groups of 15 different seaweed species. Sci Rep. (2022) 12:19610. doi: 10.1038/s41598-022-23609-8, PMID: 36380074 PMC9666456

[34] Rizvi NB Aleem S Khan MR Ashraf S Busquets RJM. Quantitative estimation of protein in sprouts of *Vigna radiata* (mung beans), *Lens culinaris* (lentils), and *Cicer arietinum* (chickpeas) by kjeldahl and Lowry methods. Molecules. (2022) 27:814. doi: 10.3390/molecules27030814, PMID: 35164080 PMC8839272

[35] Priego-Capote F de Castro MLJT. Focused microwave-assisted Soxhlet extraction: a convincing alternative for total fat isolation from bakery products. Talanta. (2005) 65:81–6. doi: 10.1016/j.talanta.2004.05.020, PMID: 18969767

[36] Kim JK Cho ML Karnjanapratum S Shin IS Sang GY. In vitro and in vivo immunomodulatory activity of sulfated polysaccharides from *Enteromorpha prolifera*. Int J Biol Macromol. (2011) 49:1051–8. doi: 10.1016/j.ijbiomac.2011.08.032, PMID: 21907732

[37] Kazharskay A Ding Y Arif M Jang F Cong Y Wang HY. Cellulose nanocrystals derived from *Enteromorpha prolifera* and their use in developing bionanocomposite films with water-soluble polysaccharides extracted from *E. prolifera*. Int J Biol Macromol. (2019) 134:390–6. doi: 10.1016/j.ijbiomac.2019.05.058, PMID: 31078599

[38] Wei H Li W Chen H Wen X He J Li J. Simultaneous Diels-Alder click reaction and starch hydrogel microsphere production via spray drying. Carbohydr Polym. (2020) 241:116351. doi: 10.1016/j.carbpol.2020.116351, PMID: 32507200

[39] Wu Y Zhou H Wei K Zhang T Che Y Nguyễn AD. Structure of a new glycyrrhiza polysaccharide and its immunomodulatory activity. Front Immunol. (2022) 13:1007186. doi: 10.3389/fimmu.2022.1007186, PMID: 36238291 PMC9551306

[40] Li J Chi Z Yu L Jiang F Liu C. Sulfated modification, characterization, and antioxidant and moisture absorption/retention activities of a soluble neutral polysaccharide from *Enteromorpha prolifera*. Int J Biol Macromol. (2017) 105:1544–53. doi: 10.1016/j.ijbiomac.2017.03.157, PMID: 28363657

[41] Jin M Wang Y Yang X Yin H Nie S Wu X. Structure characterization of a polysaccharide extracted from noni (*Morinda citrifolia* L.) and its protective effect against DSS-induced bowel disease in mice. Food Hydrocoll. (2019) 90:189–97. doi: 10.1016/j.foodhyd.2018.11.049, PMID: 40191147

[42] Xu D Wu Q Liu W Hu G Meng H Wang J. Therapeutic efficacy and underlying mechanisms of *Gastrodia elata* polysaccharides on dextran sulfate sodium-induced inflammatory bowel disease in mice: modulation of the gut microbiota and improvement of metabolic disorders. Int J Biol Macromol. (2023) 248:125919. doi: 10.1016/j.ijbiomac.2023.125919, PMID: 37481182

[43] Li Z Wen Q Pi J Zhang D Nie J Wei W. An inulin-type fructan isolated from *Serratula chinensis* alleviated the dextran sulfate sodium-induced colitis in mice through regulation of intestinal barrier and gut microbiota. Carbohydr Polym. (2023) 320:121206. doi: 10.1016/j.carbpol.2023.121206, PMID: 37659809

[44] Liu M Liu Z Zhang N Cao Z Fu J Yuan W. Preparation of polysaccharides from *Crepis tectorum* Linn. And the regulation effects on intestinal microbiota. Process Biochem. (2023) 130:50–66. doi: 10.1016/j.procbio.2023.04.004

[45] Yi J Li X Wang S Wu T Liu P. Steam explosion pretreatment of *Achyranthis bidentatae* radix: modified polysaccharide and its antioxidant activities. Food Chem. (2022) 375:131885. doi: 10.1016/j.foodchem.2021.131885, PMID: 34923399

[46] Lo CT Chang CA Chiu KH Tsay PK Jen JF. Correlation evaluation of antioxidant properties on the monosaccharide components and glycosyl linkages of polysaccharide with different measuring methods. Carbonhydr Polym. (2011) 86:320–7. doi: 10.1016/j.carbpol.2011.04.056

[47] Wang Z Zheng Y Lai Z Hu X Wang L Wang X. Effect of monosaccharide composition and proportion on the bioactivity of polysaccharides: a review. Int J Biol Macromol. (2024) 254:127955. doi: 10.1016/j.ijbiomac.2024.127955 37944714

[48] Akbari-Alavijeh S Soleimanian-Zad S Sheikh-Zeinoddin M Hashmi S. Pistachio hull water-soluble polysaccharides as a novel prebiotic agent. Int J Biol Macromol. (2018) 107:808–16. doi: 10.1016/j.ijbiomac.2017.09.049, PMID: 28928068

[49] Chen G Chen X Yang B Yu Q Wei X Ding Y. New insight into bamboo shoot (*Chimonobambusa quadrangularis*) polysaccharides: impact of extraction processes on its prebiotic activity. Food Hydrocoll. (2019) 95:367–77. doi: 10.1016/j.foodhyd.2019.04.046

[50] Cheng JJ Chao CH Chang PC Lu MK. Studies on anti-inflammatory activity of sulfated polysaccharides from cultivated fungi *Antrodia cinnamomea*. Food Hydrocoll. (2016) 48:208–12. doi: 10.1016/j.foodhyd.2015.02.034

[51] Jiang F Ding Y Tian Y Yang R Quan M Tong Z. Hydrolyzed low-molecular-weight polysaccharide from *Enteromorpha prolifera* exhibits high anti-inflammatory activity and promotes wound healing. Biomater Adv. (2022) 133:112637. doi: 10.1016/j.msec.2021.112637, PMID: 35527149

[52] Dutra NLS Brito TVD Magalhes DDA Sousa SG Barbosa ALR. Sulfated polysaccharide extracted from seaweed *Gracilaria caudata* attenuates acetic acid-induced ulcerative colitis. Food Hydrocoll. (2020) 111:106221. doi: 10.1016/j.foodhyd.2020.106221, PMID: 40191147

[53] Liu X Huang L Zhang X Xu XF. Polysaccharides with antioxidant activity: extraction, beneficial roles, biological mechanisms, structure-function relationships, and future perspectives: a review. Int J Biol Macromol. (2025) 300:140221. doi: 10.1016/j.ijbiomac.2025.140221, PMID: 39855511

[54] Meng Z Miao D Xuan-Xuan Z Ren-Ji W Yu-Ling W Bu-Ying L. 2, 3, 5, 4′-Tetrahydroxystilbene-2-O-β-D-glucoside protects hearts from isoproterenol-induced cardiac hypertrophy in mice. Chin J Extracorporeal Circulat. (2016) 137:111420. doi: 10.1016/j.cjcc.2016.11.1420

[55] Li H Chen X Liu J Chen M Huang M Huang G. Ethanol extract of *Centella asiatica* alleviated dextran sulfate sodium-induced colitis: restoration on mucosa barrier and gut microbiota homeostasis. J Ethnopharmacol. (2021) 267:113445. doi: 10.1016/j.jep.2020.113445, PMID: 33022343

[56] Xing C Wang M Ajibade AA Tan P Fu C Chen L. Microbiota regulate innate immune signaling and protective immunity against cancer. Cell Host Microbe. (2021) 29:959–974.e7. doi: 10.1016/j.chom.2021.03.016, PMID: 33894128 PMC8192480

[57] Cui L Guan X Ding W Luo Y Feng L. *Scutellaria baicalensis* Georgi polysaccharide ameliorates DSS-induced ulcerative colitis by improving intestinal barrier function and modulating gut microbiota. Int J Biol Macromol. (2020) 166:1035–45. doi: 10.1016/j.ijbiomac.2020.10.259, PMID: 33157130

[58] Huo J Wu Z Sun W Wang Z Wu J Huang M. Protective effects of natural polysaccharides on intestinal barrier injury: a review. J Agr Food Chem. (2022) 70:711–35. doi: 10.1021/acs.jafc.1c05966, PMID: 35078319

[59] Loubet Filho PS Dias TO Reis VHOT Moya AMTM Santos EF Cazarin CBB. Feed your gut: functional food to improve the pathophysiology of inflammatory bowel disease. J Funct Foods. (2022) 93:105073. doi: 10.1016/j.jff.2022.105073, PMID: 40191147

[60] Zhai Z Zhang F Cao R Ni X Xin Z Deng J. Cecropin a alleviates inflammation through modulating the gut microbiota of C57BL/6 mice with DSS-induced IBD. Front Microbiol. (2019) 10:1595. doi: 10.3389/fmicb.2019.01595, PMID: 31354682 PMC6635700

[61] Morgan XC Tickle TL Sokol H Gevers D Devaney KL Ward DV. Dysfunction of the intestinal microbiome in inflammatory bowel disease and treatment. Genome Biol. (2012) 13:R79. doi: 10.1186/gb-2012-13-9-r79, PMID: 23013615 PMC3506950

[62] Fernández J Redondo-Blanco S Gutiérrez-del-Río I Miguélez EM Villar CJ Lombó F. Colon microbiota fermentation of dietary prebiotics towards short-chain fatty acids and their roles as anti-inflammatory and antitumour agents: a review. J Funct Foods. (2016) 25:511–22. doi: 10.1016/j.jff.2016.06.032

[63] Kang Y Yang C Zhang S Ross CF Zhu MJ. Goji berry modulates gut microbiota and alleviates colitis in IL-10-deficient mice. Mol Nutr Food Res. (2018) 62:e1800535. doi: 10.1002/mnfr.201800535, PMID: 30243032

[64] Mukhopadhya I Hansen R El-Omar EM Hold GL. IBD—what role do Proteobacteria play? Nat Rev Gastroenterol Hepatol. (2012) 9:219–30. doi: 10.1038/nrgastro.2012.14, PMID: 22349170

[65] Seishima J Iida N Kitamura K Yutani M Kaneko S. Gut-derived *Enterococcus faecium* from ulcerative colitis patients promotes colitis in a genetically susceptible mouse host. Genome Biol. (2019) 20:252. doi: 10.1186/s13059-019-1879-9, PMID: 31767028 PMC6876129

[66] Swanson L Katkar GD Tam J Pranadinata RF Chareddy Y Coates J. TLR4 signaling and macrophage inflammatory responses are dampened by GIV/Girdin. Proc Natl Acad Sci. (2020) 117:26895–906. doi: 10.1073/pnas.2011667117, PMID: 33055214 PMC7604444

[67] Zhu C Song K Shen Z Quan Y Tan B Luo W. *Roseburia intestinalis* inhibits interleukin-17 excretion and promotes regulatory T cells differentiation in colitis. Mol Med Rep. (2018) 17:7567–74. doi: 10.3892/mmr.2018.8833, PMID: 29620246 PMC5983956

[68] Liu Z Liu F Wang W Sun C Gao D Ma J. Study of the alleviation effects of a combination of *lactobacillus rhamnosus* and inulin on mice with colitis. Food Funct. (2020) 11:3823–37. doi: 10.1039/c9fo02992c, PMID: 32329478

[69] Derrien M Vaughan EE Plugge CM de Vos WM. *Akkermansia muciniphila* gen. Nov., sp. nov., a human intestinal mucin-degrading bacterium. Int J Syst Evol Microbiol. (2004) 54:1469–76. doi: 10.1099/ijs.0.02873-0, PMID: 15388697

[70] Etienne-Mesmin L Chassaing B Desvaux M De Paepe K Gresse R Sauvaitre T. Experimental models to study intestinal microbes–mucus interactions in health and disease. FEMS Microbiol Rev. (2019) 43:457–89. doi: 10.1093/femsre/fuz013, PMID: 31162610

[71] Plovier H Everard A Druart C Depommier C Van Hul M Geurts L. A purified membrane protein from *Akkermansia muciniphila* or the pasteurized bacterium improves metabolism in obese and diabetic mice. Nat Med. (2017) 23:107–13. doi: 10.1038/nm.4236, PMID: 27892954

[72] Zhang J Ni Y Qian L Fang Q Zheng T Zhang M. Decreased abundance of *Akkermansia muciniphila* leads to the impairment of insulin secretion and glucose homeostasis in lean type 2 diabetes. Adv Sci. (2021) 8:2100536. doi: 10.1002/advs.202100536, PMID: 34085773 PMC8373164

