# Supplementary material for: Enteromorpha prolifera soluble dietary fiber alleviates ulcerative colitis through restoration of mucosal barrier and gut microbiota homeostasis
Source: Front Nutr. 2025 Apr 24;12:1579889. doi: 10.3389/fnut.2025.1579889 (PMC12058658; doi:10.3389/fnut.2025.1579889)
Supplement: Supplementary file 1 [file Table_1.docx]

Supplementary Material

*Entermorpha prolifera* soluble dietary fiber alleviates ulcerative colitis through restoration of mucosal barrier and gut microbiota homeostasis

**Yuan-Yuan Ding^2, #^, Xin-Yi Tang^1, #^, Yu Tian^1^, Feng-Long Zhang^1^, Xiang Ding^1^, Meng-Chun Qi^2^, Wei Dong^2^, Chen-Guang Liu^1,^ ***

^1^ College of Marine Life Sciences, Ocean University of China, No. 5 Yushan Road, 266003 Qingdao, China

^2^ College of Stomatology, North China University of Science and Technology, 063210 Tangshan, China

* Corresponding authors.

^#^ These authors contributed equally to this work

E-mail address: liucg@ouc.edu.cn (C. Liu)

# Supplementary Figures and Tables

## Supplementary Figures

**
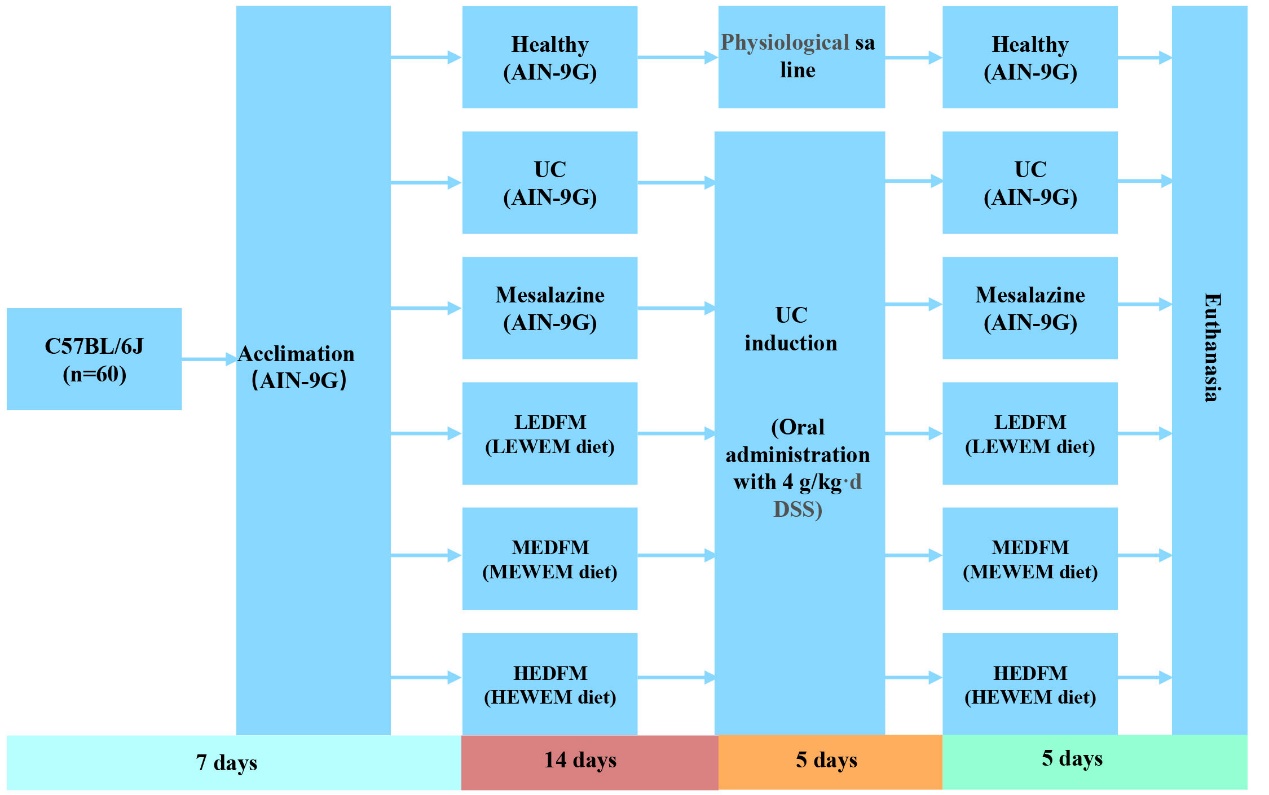
**

**Fig. S1.** The induction and EDFM dietary treatment regimen of DSS-induced UC

**
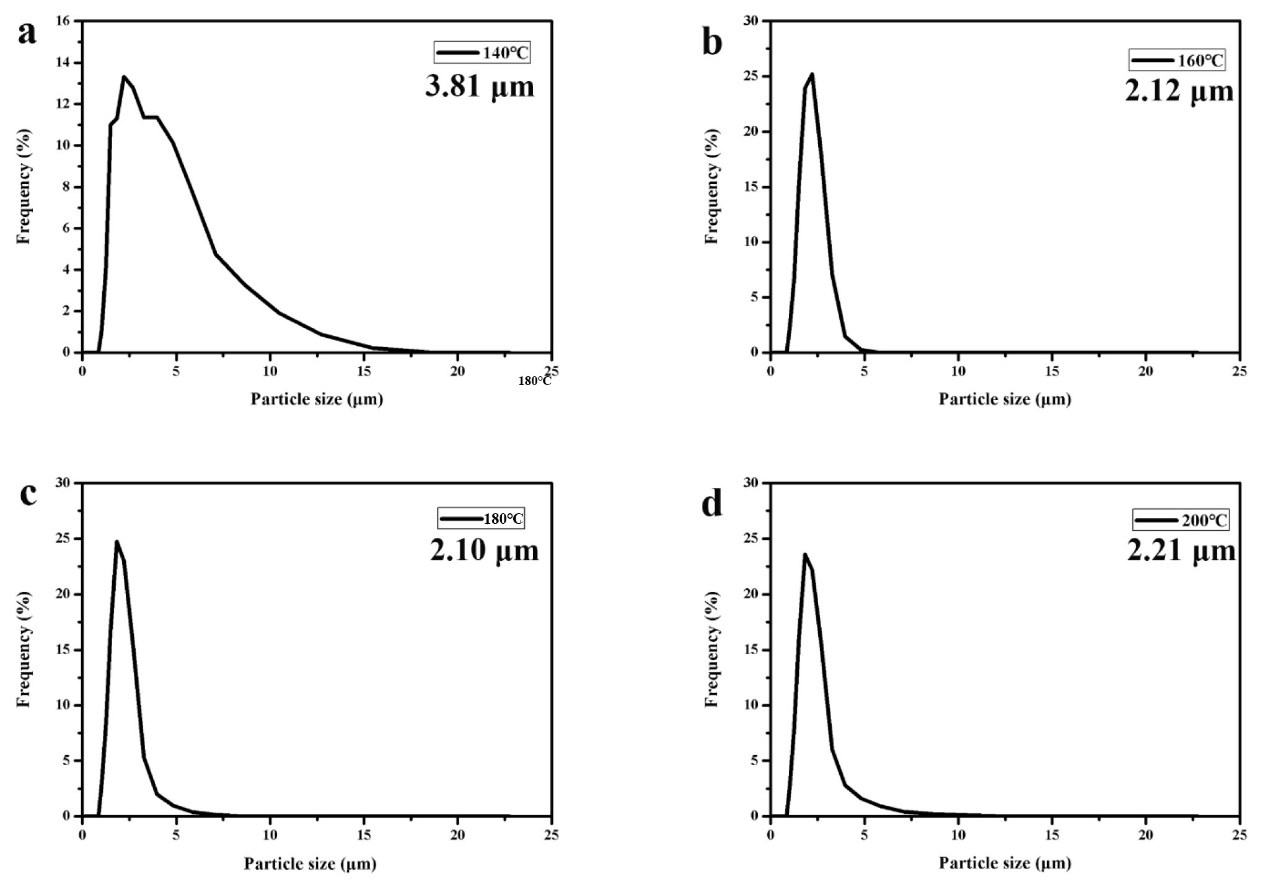
**

**Fig. S2.** Effects of inlet temperature on the particle size of EDFM. (a) 140 ℃. (b) 160 ℃. (c) 180 ℃. (d) 200 ℃

**
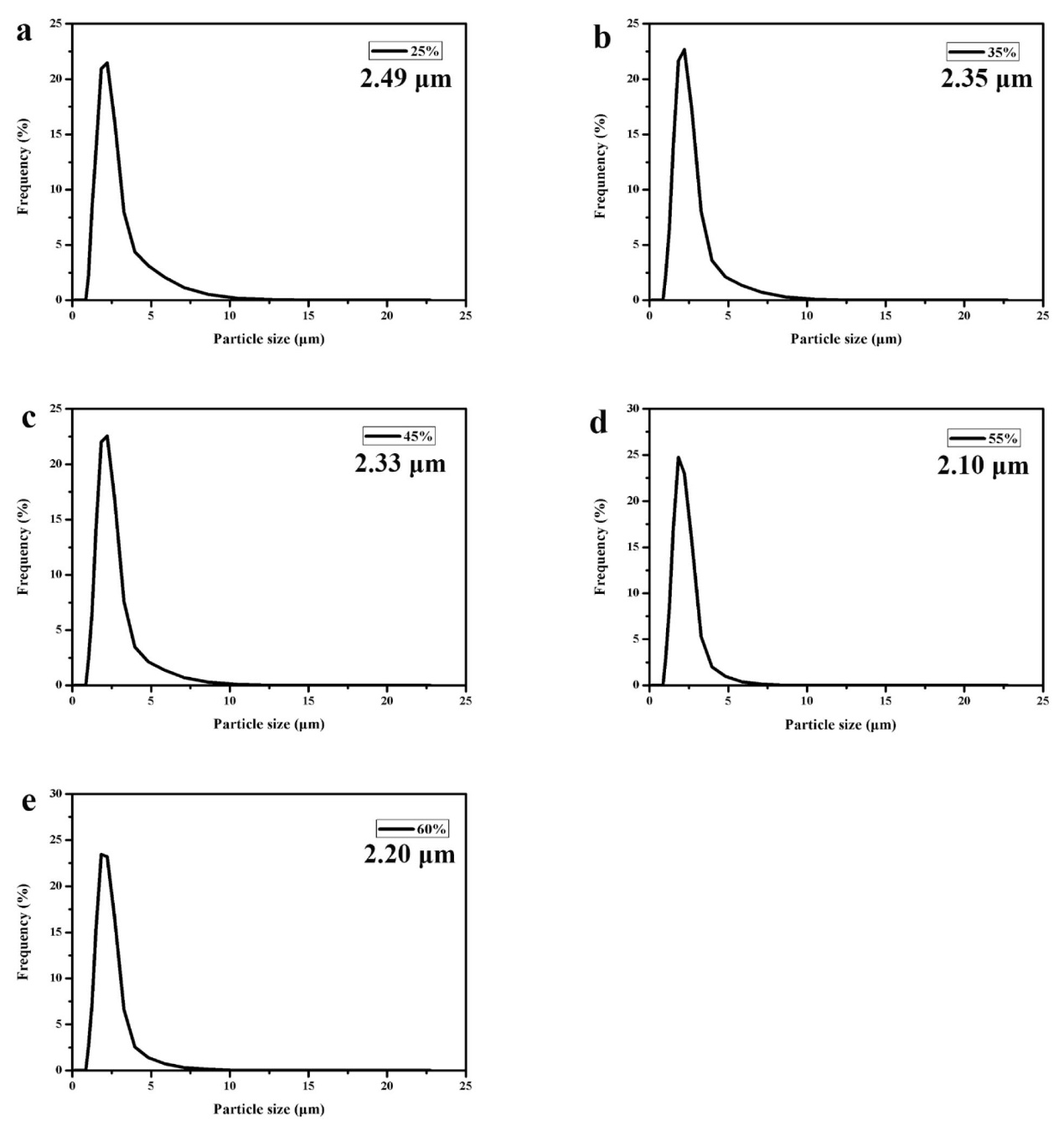
**

**Fig. S3.** Effects of injection rate on the particle size of EDFM. (a) 25%. (b) 35%. (c) 45%. (d) 55%. (e) 60%.

**
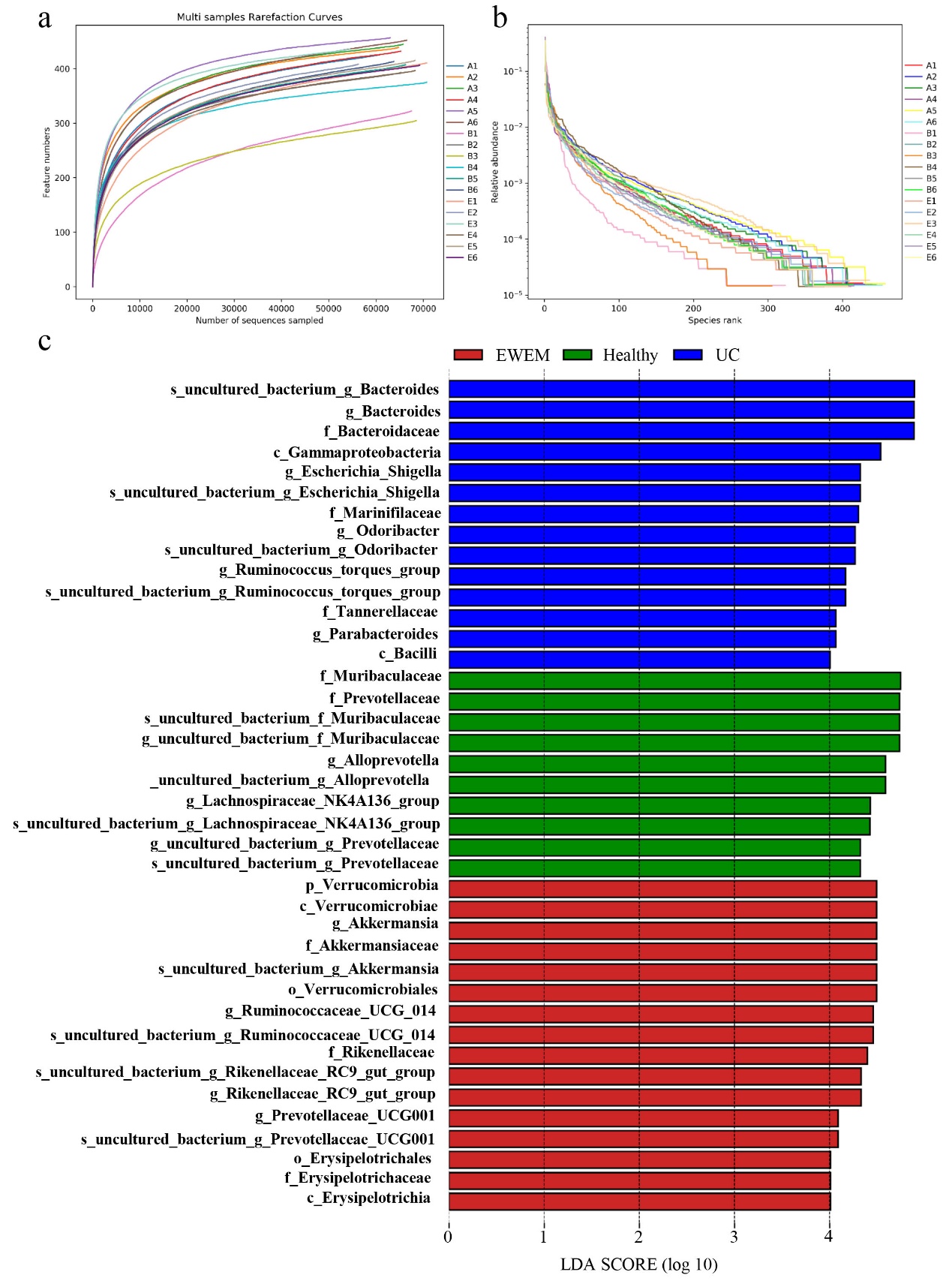
**

**Fig. S4.** (a) The rarefaction curve and (b) rank abundance curve of each sample. A1-A6 represent six samples of the mice in the Healthy group. B1-B6 represent six samples of the mice in the UC group. C1-C6 represent six samples of the mice in the EDFM group. (c) Histogram of LDA value distribution of the differential microbial community among the Healthy, UC and EDFM groups.

## Supplementary Tables

**Table S1.** The scoring table of the body weight, stool characters and fecal occult blood of mice

| Score | Body weight (%) | Stool characters | Fecal occult blood |
| --- | --- | --- | --- |
| 0 | - | Normal | Negative（-） |
| 1 | 1-5 | Loosely formed feces that do not stick to the anus | Positive（±） |
| 2 | 5-10 | Loosely formed feces that do not stick to the anus | Positive（+） |
| 3 | 10-15 | Loosely formed feces that stick to the anus | Positive（++） |
| 4 | >15 | Diarrhea | Bloody stool（>++） |

**Table S2.** Histopathological scoring criteria of the colon tissues

| The colon histopathologic appearance. | Score |
| --- | --- |
| No obvious inflammatory reaction | 0 |
| Low-level inflammatory cell infiltration; Without destruction of intestinal mucosa | 1 |
| Moderate inflammatory infiltration; The intestinal recess deepened and the intestinal wall thickened, but did not invade the muscular layer | 2 |
| Severe inflammatory infiltration; Vascular hyperplasia; Intestinal wall thickened and invaded the muscular layer | 3 |
| Massive inflammatory infiltration; The decrease of goblet cells; Vascular hyperplasia; Intestinal wall thickened and invaded the muscular layer | 4 |

**Table S3.** The yield of EDFM under various preparation conditions of spay dryer

| Factors | | Yield (%) |
| --- | --- | --- |
| Inlet temperature (℃) | 140 | 45.3 ± 2.4 |
|  | 160 | 63.2 ± 3.6 |
|  | 180 | 76.9 ± 4.1 |
|  | 200 | 77.2 ± 3.9 |
| Injection rate (%) | 25 | 74.2 ± 2.7 |
|  | 35 | 75.9 ± 2.5 |
|  | 45 | 74.1 ± 1.9 |
|  | 55 | 65.3 ± 3.8 |
|  | 60 | 60.8 ± 1.6 |
